# Osteoporosis and skeletal dysplasia caused by pathogenic variants in *SGMS2*

**DOI:** 10.1172/jci.insight.126180

**Published:** 2019-04-04

**Authors:** Minna Pekkinen, Paulien A. Terhal, Lorenzo D. Botto, Petra Henning, Riikka E. Mäkitie, Paul Roschger, Amrita Jain, Matthijs Kol, Matti A. Kjellberg, Eleftherios P. Paschalis, Koen van Gassen, Mary Murray, Pinar Bayrak-Toydemir, Maria K. Magnusson, Judith Jans, Mehran Kausar, John C. Carey, Pentti Somerharju, Ulf H. Lerner, Vesa M. Olkkonen, Klaus Klaushofer, Joost C.M. Holthuis, Outi Mäkitie

**Affiliations:** 1 Folkhälsan Institute of Genetics, Folkhälsan Research Center, Helsinki, Finland, and Research Program for Clinical and Molecular Metabolism, Faculty of Medicine, University of Helsinki, Finland.; 2 Children’s Hospital, University of Helsinki and Helsinki University Hospital, Helsinki, Finland.; 3 Department of Genetics, University Medical Center Utrecht, Utrecht, Netherlands.; 4 Division of Medical Genetics, Department of Pediatrics, University of Utah, Salt Lake City, Utah, USA.; 5 Centre for Bone and Arthritis Research, Department of Internal Medicine and Clinical Nutrition, Institute for Medicine, Sahlgrenska Academy, University of Gothenburg, Gothenburg, Sweden.; 6 Ludwig Boltzmann Institute of Osteology at the Hanusch Hospital of WGKK and AUVA Trauma Centre Meidling, 1st Medical Department, Hanusch Hospital, Vienna, Austria.; 7 Molecular Cell Biology Division, Department of Biology/Chemistry, University of Osnabrück, Osnabrück, Germany.; 8 Department of Biochemistry and Developmental Biology, Faculty of Medicine, University of Helsinki, Helsinki, Finland.; 9 Department of Genetics, University Medical Center Utrecht, Utrecht, Netherlands.; 10 Division of Pediatric Endocrinology & Diabetes, Department of Pediatrics, University of Utah, Salt Lake City, Utah, USA.; 11 Department of Pathology, University of Utah, Salt Lake City, Utah, USA, and ARUP Laboratories, Salt Lake City, Utah, USA.; 12 Department of Microbiology and Immunology, Institute for Biomedicine, Sahlgrenska Academy, University of Gothenburg, Gothenburg, Sweden.; 13 Laboratory of Metabolic Diseases, University Medical Center Utrecht, Utrecht, Netherlands.; 14 Minerva Foundation Institute for Medical Research, Biomedicum, Helsinki, Finland, and Department of Anatomy, Faculty of Medicine, University of Helsinki, Helsinki,Finland.; 15 Biochemistry and Biophysics Division, Bijvoet Center and Institute of Biomembranes, Utrecht University, Utrecht, Netherlands.; 16 Department of Molecular Medicine and Surgery, Karolinska Institutet, and Clinical Genetics, Karolinska University Laboratory, Karolinska University Hospital, Stockholm, Sweden.

**Keywords:** Endocrinology, Genetics, Bone disease, Genetic diseases, Osteoporosis

## Abstract

Mechanisms leading to osteoporosis are incompletely understood. Genetic disorders with skeletal fragility provide insight into metabolic pathways contributing to bone strength. We evaluated 6 families with rare skeletal phenotypes and osteoporosis by next-generation sequencing. In all the families, we identified a heterozygous variant in *SGMS2*, a gene prominently expressed in cortical bone and encoding the plasma membrane–resident sphingomyelin synthase SMS2. Four unrelated families shared the same nonsense variant, c.148C>T (p.Arg50*), whereas the other families had a missense variant, c.185T>G (p.Ile62Ser) or c.191T>G (p.Met64Arg). Subjects with p.Arg50* presented with childhood-onset osteoporosis with or without cranial sclerosis. Patients with p.Ile62Ser or p.Met64Arg had a more severe presentation, with neonatal fractures, severe short stature, and spondylometaphyseal dysplasia. Several subjects had experienced peripheral facial nerve palsy or other neurological manifestations. Bone biopsies showed markedly altered bone material characteristics, including defective bone mineralization. Osteoclast formation and function in vitro was normal. While the p.Arg50* mutation yielded a catalytically inactive enzyme, p.Ile62Ser and p.Met64Arg each enhanced the rate of de novo sphingomyelin production by blocking export of a functional enzyme from the endoplasmic reticulum. *SGMS2* pathogenic variants underlie a spectrum of skeletal conditions, ranging from isolated osteoporosis to complex skeletal dysplasia, suggesting a critical role for plasma membrane–bound sphingomyelin metabolism in skeletal homeostasis.

## Abstract 2

The identification of 6 families with childhood-onset osteoporosis with mutations in SGMS2 suggests a critical role for plasma membrane–bound sphingomyelin metabolism in skeletal homeostasis.

## Introduction

Osteoporosis is a major clinical and public health concern with significant morbidity, disability, and mortality (1) The underlying causes remain incompletely understood. Rare inherited skeletal disorders have often shed light on the genetic and molecular mechanisms governing bone health (2). Several such rare disorders are associated with early-onset osteoporosis, with or without additional clinical traits. One example is osteoporosis with calvarial doughnut lesions (CDLs) (Online Mendelian Inheritance in Man [OMIM] #126550). This autosomal dominant skeletal disorder is characterized by low-bone mineral density, increased spinal and peripheral fractures, and sclerotic, doughnut-shaped lesions in the cranial bones. In the current nosology of skeletal disorders (3), CDL is included with osteogenesis imperfecta (OI) in the group of bone fragility disorders. The genetic cause of CDL has remained unknown.

Herein, we describe 6 families with a total of 13 subjects affected by early-onset osteoporosis of varying severity and a spectrum of other features, ranging from mild cranial sclerotic lesions characteristic of CDL to spondylometaphyseal dysplasia with marked cranial sclerosis. In all affected subjects, we identified potentially novel heterozygous pathogenic variants in the sphingomyelin synthase 2 gene (*SGMS2*) (NM_001136257, uniprot: Q8NHU3). We performed functional studies to address the pathogenic role of these variants. Our findings underscore the importance of intact plasma membrane–bound sphingomyelin metabolism for normal bone mineralization and bone strength.

## Results

### Patients

**Figure 1:**
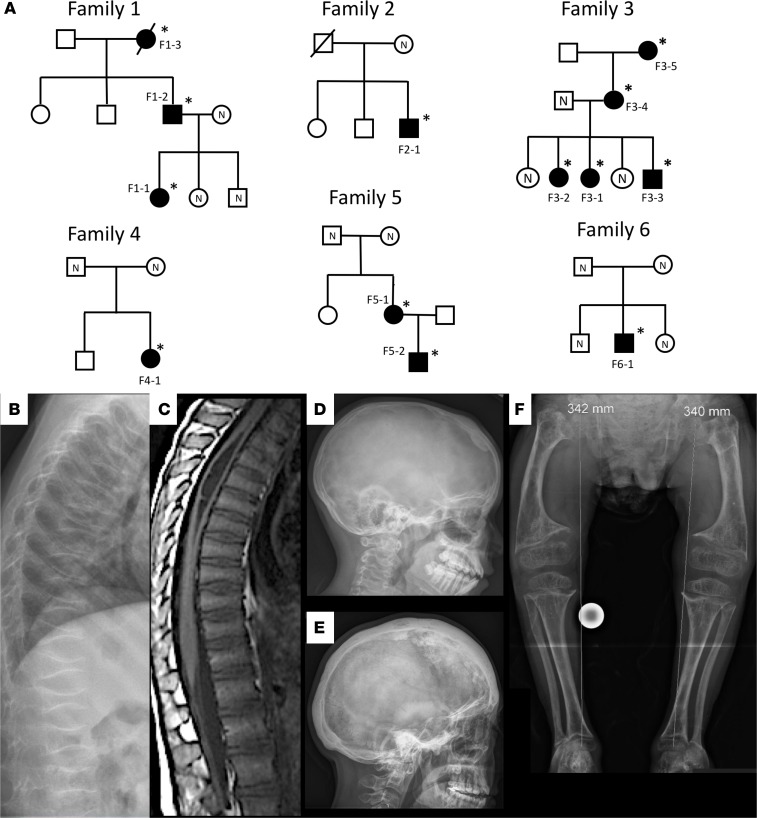
Characteristics of the 6 families with osteoporosis and a heterozygous *SGMS2* mutation. (**A**) Pedigrees of the 6 families. Squares represent males, circles represent females, black symbols represent affected family members, white symbols represent unaffected family members, and slashes deceased family members. Individuals with a confirmed mutation are marked with an asterisk, and mutation-negative individuals are marked with N. Codes refer to individual data in the text and in Tables 1 and 2. (**B**) Patient 4-1 with a p.Arg50* mutation has at 6 years severe osteoporosis with spinal compression fractures involving all vertebrae. (**C**) Patient F3-1, with the same p.Arg50* mutation, has at 7 years on spinal magnetic resonance imaging multiple compression fractures involving nearly every thoracic and lumbar vertebra and moderate syringomyelia of the upper thoracic spine involving levels T3-T8. (**D**) Patient F3-2 has at 13 years sclerotic lesions in the left frontal bone and the right parietal bone. (**E**) Patient F5-1 with the Ile62Ser mutation has at adult age severe skull hyperostosis with sclerotic and lytic lesions throughout the skull. (**F**) Patient F6-1 with the p.Met64Arg mutation has at 11 years severe spondylometaphyseal dysplasia with poorly mineralized bones, short and wide long bones with metaphyseal widening, abnormal bone texture, and bilateral coxa vara.

Through international collaboration we recruited 6 families (Figure 1A) to the study. The recruitment occurred by two means: (a) by asking clinicians about phenotypically matching patients with the combination of osteoporosis or fractures and sclerotic cranial lesions, similar to that described in CDL, and, (b) after identification of the gene defect, by genetic and phenotypic match through GeneMatcher (4). The clinical data are summarized in Tables 1 and 2 and presented here separately for each family.

#### Family 1.

This Finnish 3-generation family with 3 affected individuals has been previously described (5). The index (F1-1) is presently a 23-year-old female with a history of spinal and peripheral fractures since childhood, low-bone mineral density, sclerotic calvarial lesions, recurrent idiopathic peripheral facial nerve palsies (Bell’s palsy), and congenital bilateral glaucoma. Her 59-year-old father (F1-2) has a similar skeletal phenotype and transient facial, trochlear, and oculomotor nerve palsies with slow recovery; their cause remained unclear despite thorough neurological evaluations. The father’s mother (F1-3), who died at age 85 years, had severe osteoporosis with sclerotic calvarial lesions, diagnosis of Alzheimer’s disease, transient peripheral facial nerve palsies and episodes of abducens and oculomotor nerve palsies, and chronic congestive glaucoma.

#### Family 2.

The index (F2-1) is a 27-year-old Finnish male with severe childhood-onset primary osteoporosis and a history of 9 low-energy peripheral fractures, multiple vertebral fractures, and a sclerotic cranial lesion. He has had no nerve palsies or ophthalmological concerns.

#### Family 3.

The index subject (F3-1) is an 8-year-old White girl from the US with severe early-onset osteoporosis, vertebral fractures involving nearly all vertebrae, mild thoracic syringomyelia, mild short stature, and no sclerotic cranial lesions. Her older sister (F3-2) has a similar clinical history but also sclerotic cranial lesions. Her younger brother (F3-3) has, at 4 years of age, low-bone mineral density but no history of fractures and no calvarial lesions. Their mother (F3-4), age 38 years, has severe osteoporosis with multiple vertebral and peripheral fractures. The mother’s mother (F3-5) has a history of osteoporosis with multiple fractures, cranial sclerotic lesions, and episodes of transient unilateral facial nerve palsies, affecting both the upper and lower face, with recovery within a month.

#### Family 4.

The index patient (F4-1), the second child of healthy White parents of Northern European ancestry, is a 6-year-old girl with severe vertebral compression fractures and a history of 6 long-bone fractures. The skull radiograph at 6 years of age was normal. She has not had neurological symptoms. Bisphosphonate treatment resulted in notable improvement in back pain and quality of life.

#### Family 5.

The index patient (F5-1) is a 44-year-old Dutch female who presented shortly after birth with a clavicular fracture and bilateral femoral fractures. She underwent femoral osteotomy at 2.5 years and intramedullary rodding at 15 years but continued to have multiple fractures. She had recurrent transient facial nerve paralysis, recovering each time within 4–6 weeks, at age 13, 20, and 41 years; unexplained transient diplopia at 30 years; and unilateral transient ptosis at 44 years. She experienced progressive hearing loss after 25 years of age. Since adulthood she has suffered from progressive ataxia due to sensory neuronopathy. Her son (F5-2) had a clavicular fracture after birth and a femoral fracture at 8 months. Bisphosphonate treatment was commenced at 2.6 years because of a radius fracture, lower limb bowing, and platyspondyly and discontinued at age 6 years; no new fractures have occurred thereafter. At 8 years of age, he was diagnosed with transient peripheral facial nerve palsy.

#### Family 6.

The index (F6-1) is an 11-year-old boy of Hispanic ancestry with severe early-onset osteoporosis, with multiple long bone fractures beginning at birth. The radiographic features evolved over time to spondylometaphyseal dysplasia with marked short stature and marked scoliosis. He had normal skull radiographs at birth but developed progressive severe cranial sclerosis. Additional findings included sensorineural hearing loss, myopia, dilated aortic root, and hypotonia with abnormal myopathic electromyography but normal muscle biopsy. He was also diagnosed by a pediatric neurologist with decreased bulbar function (facial diplegia) at 5 years, which was no longer present in neurological evaluation at 11 years. He underwent several courses of bisphosphonate treatment from infancy to age 4 years and has not had further long-bone fractures.

### Identification of *SGMS2* mutations

We arrived at the genetic conclusions independently in each family using next-generation sequencing. Whole-exome sequencing (WES) was performed for family 1 at Institute for Molecular Medicine Finland (FIMM) (Helsinki, Finland), for families 3, 4, and 6 at the University of Utah, and family 5 at University Medical Center Utrecht. Whole-genome sequencing was performed for the index subject in family 2 at the Science for Life Laboratory. All genetic findings were confirmed by Sanger sequencing.

We identified heterozygous *SGMS2* variants in 13 affected subjects from 6 unrelated families altogether (Figure 1A and Supplemental Figure 1; supplemental material available online with this article; https://doi.org/10.1172/jci.insight.126180DS1). In family 1, exome sequencing identified a c.148C>T variant, which introduces a premature stop codon in exon 2 (p.Arg50*). The same variant was detected in affected individuals in families 2 and 3. In family 4, the same c.148C>T variant was identified de novo. In family 5, exome sequencing identified a de novo c.185T>G (p.Ile62Ser) variant in the affected mother; her affected son harbored the same missense variant. In family 6, exome sequencing identified a de novo c.191T>G (p.Met64Arg) variant in the index. All these variants were absent in databases (dbSNP, ExAC, gnomAD) and were not found in >180 Finnish controls by Sanger sequencing.

### Skeletal characteristics in patients with *SGMS2* pathogenic variants

**Figure 2:**
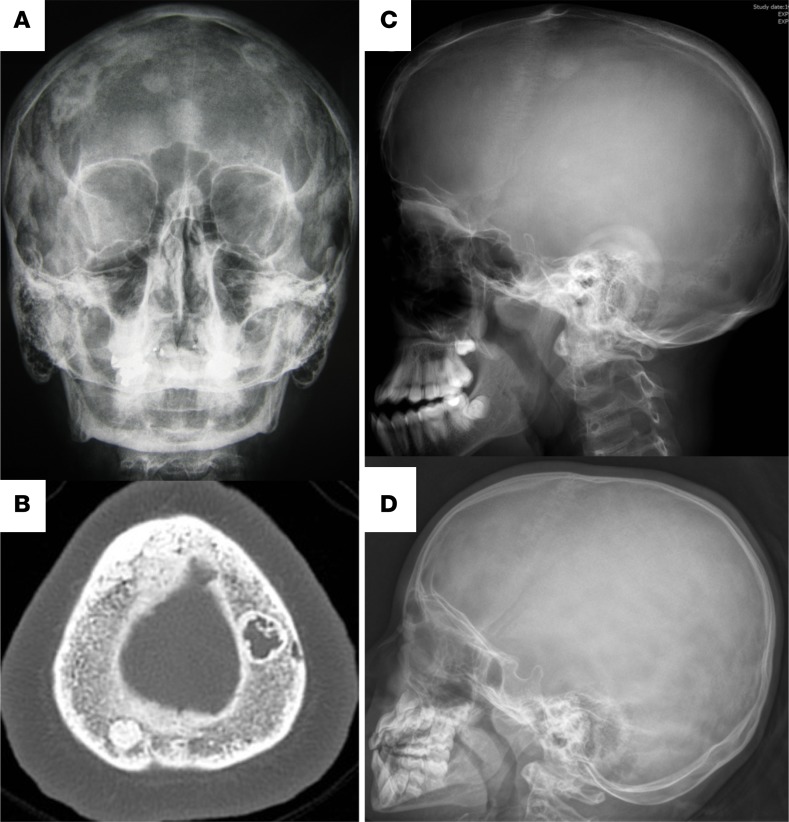
Characteristics of the skull lesions. (**A**) Skull radiographs of 3 patients with the p.Arg50* mutation showing extensive sclerotic lesions in patient 1-2 at adult age. (**B**) On computerized tomography, the lesions have mixed sclerotic and lytic appearance. Patient F2-1 has only one single sclerotic lesion at the age of 15 years (**C**) while patient F4-1 has normal skull radiograph at 6 years (**D**).

**Figure 3:**
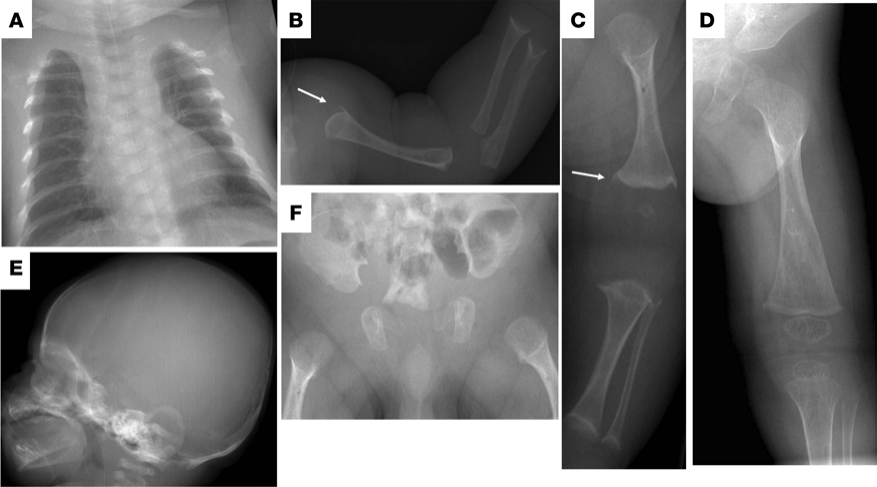
Skeletal characteristics. Radiographs of patient F5-2 with the p.Ile62Ser mutations at age 1–4 months showing thin ribs (**A**), abnormally short, wide, and poorly mineralized long bones in the arm (**B**) and leg (**C**). The proximal humerus and distal femur have abnormal metaphyses with pikes (arrows) reminiscent of those seen in hypophosphatasia. At 4 months, the long bones are poorly mineralized and show marked undertubulation with metaphyseal widening of the distal femur (**D**). Skull is osteopenic and does not show wormian bones or sclerotic lesions (**E**). Pelvis shows undermineralization but regular iliac wings and abnormally wide and poorly mineralized proximal femurs (**F**).

The affected subjects shared several skeletal findings, including low-bone mineral density combined with increased peripheral and/or spinal fractures, often but not always associated with sclerotic cranial lesions (Tables 1 and 2, Figures 1 and 2, and Supplemental Figure 2). The skeletal findings varied in severity: the 10 patients with *SGMS2* variant p.Arg50* had a milder phenotype, with increased bone fragility but without major deformities or height deficit (Table 1; Figure 1, B–D; and Supplemental Figure 2). In contrast, the 2 subjects with p.Ile62Ser and 1 subject with p.Met64Arg had multiple spinal and long-bone fractures of neonatal onset, severe short stature, and radiographic evidence of spondylometaphyseal dysplasia (metaphyseal changes associated with marked platyspondyly and scoliosis). Furthermore, the degree of cranial sclerosis was considerably greater in these patients (Table 2; Figure 1, E and F; Figure 3; and Supplemental Figure 3). Biochemical parameters were mostly normal, but alkaline phosphatase and other bone turnover markers tended to be elevated (Supplemental Tables 1 and 2).

**Figure 4:**
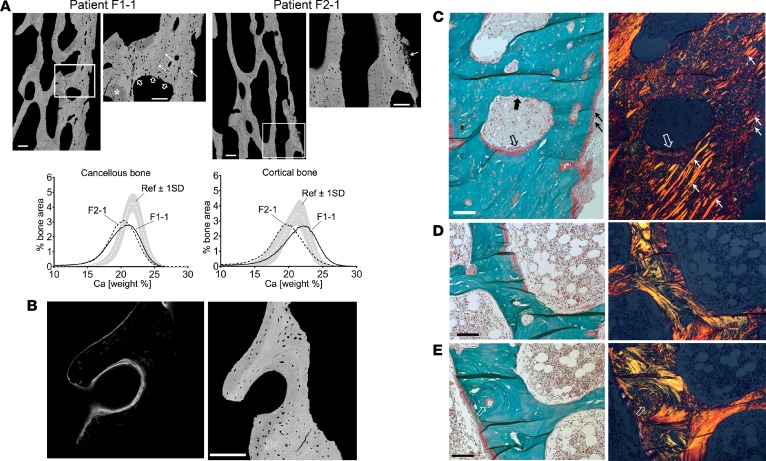
Bone tissue characteristics. (**A**) Quantitative backscatter electron imaging (qBEI) of the transiliac bone biopsy samples. Top: There is no clear separation between the trabecular and cortical compartment. Pixel gray levels correspond to mineral content (bright to higher and dark to lower). Scale bars: 200 μm; 100 μm (zoom). Nonmineralized Sharpey’s fibres are infusing the cortex (solid white arrows, zoomed-in areas), clearly identified by polarized light microscopy (see **C**). Bottom: Bone mineralization density distribution (BMDD) curves for the 2 patients. Ref, reference BMDD for children (66). BMDD for cancellous bone showed reduced mineral content, increased heterogeneity in matrix mineralization, and highly increased portion of lowly mineralized bone. In cortical bone of patient F1-1, the BMDD showed a peak shift to higher mineralization due to increased proportion of primary bone mineralized to a higher extent. In patient F2-1, the cortical bone mineralization was overall reduced. (**B**) Confocal laser scanning microscopy (CLSM) of fluorescence labeled bone. Left: CLSM image of a mineralizing surface shows diffuse appearance of double labeling. Right: Corresponding qBEI image (identical bone area; scale bar: 200 μm). Of note, the osteocyte lacunae are enlarged and of abnormal shape. (**C**-**E**) Polarized light microscopy of the bone samples. Paired images of bright-field (right) and linear polarized (left) light (scale bars: 100 μm). (**C**) Cortical detail of patient F1-1 (region close to zoomed-in area of **A**): predominantly woven bone infused with Sharpey’s fibres (thin white and black arrows) and bone formation (thick empty black and white arrows) and resorption (thick black arrow in **C** and thick empty white arrows in **A**). Of note, the new osteoid does not show a lamellar fibril arrangement. (**D** and **E**) Examples of bone tissue with coexisting woven and lamellar bone matrix. Of note, an osteon (arrows) has woven character in the center though at the peripheral region it is lamellar.

To gain further insight into bone tissue characteristics, we reevaluated transiliac bone biopsy samples from patient F1-1 and patient F2-1, obtained at age 12 and 15 years at Children’s Hospital, University of Helsinki, using identical tetracycline labeling and sample preparation protocols, and a femoral bone biopsy from patient F5-1, obtained at age 15 years during a surgery to correct femoral bowing by osteotomy followed by intramedullar rodding. The biopsy was taken at the osteotomy area and far from the growth plate without a preceding tetracycline labeling. The transiliac biopsies showed variable outcomes (Supplemental Table 3): bone volume was normal in patient F1-1 and reduced in patient F2-1. In both patients, trabecular thickness was decreased, and cortical bone was reduced in thickness and showed remarkable trabecularization (Figure 4). Patient F2-1 had highly increased osteoid volume, thickness, and surface, while these parameters were reduced in patient F1-1. Parameters of bone resorption indicated increased osteoclast numbers in both patients (Supplemental Table 3). Polarized light microcopy revealed in all 3 samples that bone matrix was predominately formed by haphazard arrangement of collagen fibrils (woven bone), while a regular lamellar arrangement of collagen fibrils characteristic for normal mature bone was sparse (Figure 4 and Supplemental Figure 4). Moreover, numerous parallelly oriented birefringent fibers (Sharpey’s fibers) interfusing the cortical bone were seen in patient F1-1 (Figure 4).

In transiliac bone biopsies bone mineralization density distribution (BMDD) for cancellous bone showed distinctly reduced mineral content, increased heterogeneity in matrix mineralization, and a highly increased portion of lowly mineralized bone in both patients compared with reference data (Figure 4, Supplemental Figure 5, and Supplemental Table 4). The BMDD of cortical bone showed a peak shift to higher mineralization in patient F1-1, while in patient F2-1 the BMDD was shifted toward reduced bone matrix mineralization, as in trabecular bone. The increase of cortical mineralization in patient F1-1, however, coincided with the appearance of Sharpey’s fibers interfusing highly mineralized primary bone, indicating that at this cortical site ligaments or tendons were adhering (Figure 4, Supplemental Figure 5, and Supplemental Table 4). Details of the bone tissue at higher magnification further indicated that osteocyte lacunae were larger and more irregular in shape than in normal mature lamellar bone (Figure 4). Raman microspectroscopic analyses suggested decelerated mineral accumulation kinetics and reduced pyridinoline, a cross-linking compound of mature collagen fibers (Supplemental Figure 6).

### *SGMS2* is prominently expressed in cortical bone

**Figure 5:**
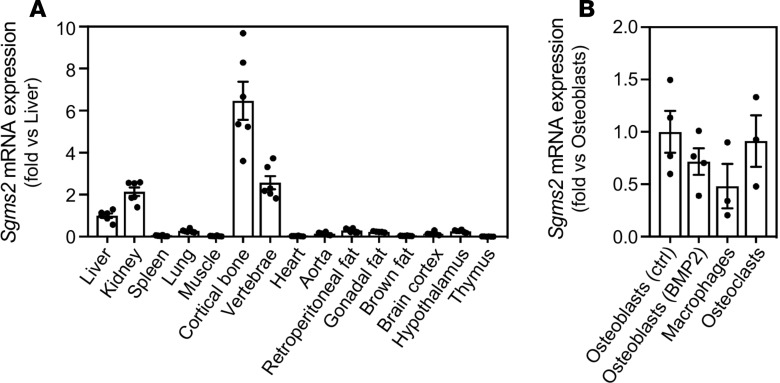
Expression of *Sgms2* in tissues and cells. (**A**) Gene expression of *Sgms2* in different tissues from C57BL/6N female mice. (**B**) Gene expression of *Sgms2* in in vitro–cultured murine primary calvarial osteoblast, cultured in the absence or presence of BMP2, and in cultured murine bone marrow–derived macrophages cultured in M-CSF and osteoclasts cultured in M-CSF and RANKL. Data are displayed as fold change versus liver in **A**, and versus osteoblasts (ctrl) in **B** ± SEM.

We evaluated mRNA expression of *Sgms2* in a murine tissue panel and found the highest expression in cortical bone, followed by vertebrae, kidney, and liver (Figure 5A) Expression levels were very low in spleen, muscle, heart, brown fat, and thymus. These results are in line with a bone-critical function of SMS2. In vitro–cultured primary murine osteoblasts, bone marrow macrophages, and osteoclasts all expressed *Sgms2* at similar levels (Figure 5B).

### Effect of *SGMS2* pathogenic variants on osteoclast differentiation and function

Because the patients’ imaging studies and transiliac bone biopsies suggested that osteoclast numbers might be increased, a finding previously reported in CDL (6), we evaluated osteoclast formation and function using peripheral blood monocytes from 2 patients with the p.Arg50* mutation (F1-1 and F2-1). The details of these experiments are presented in the Supplemental Methods. Both patients had normal levels and proportions of peripheral blood monocytes but an increased proportion of monocytes with surface expression of M-CSF receptor and vitronectin receptor, both of which are required for normal osteoclastogenesis (7–9) (Supplemental Figure 7). When CD14^+^ monocytes were cultured in the presence of M-CSF and RANKL to induce osteoclast differentiation (10), the *SGMS2* mutation had no effect on osteoclast morphology (Supplemental Figure 8). On plastic, at optimal concentration of RANKL, no obvious differences between patients and controls were seen (Supplemental Figure 9). When monocytes were differentiated in the presence of M-CSF and RANKL on bone discs, the patients’ osteoclasts had a similar morphology as the controls and formed resorption pits and actin rings (Supplemental Figure 8). The resorptive capacity showed no consistent differences from the controls (Supplemental Figure 10). Increased expression of M-CSF receptor could indicate that monocytes are more prone to M-CSF–induced expansion of a cell lineage, which can give rise to macrophages, osteoclast progenitors, and dendritic cells. The increased expression of integrin suggests that osteoclasts could be more active in bone resorption (11, 12), but this could not be confirmed by measuring osteoclast activity in vitro.

### Effect of *SGMS2* pathogenic variants on blood metabolites

Untargeted metabolomics using direct-infusion high-resolution mass spectrometry in dried bloodspots of 12 patients did not reveal any hydrophilic small-molecule biomarkers associated with the *SGMS2* variants (data not shown). Determination of serum sphingosine-1-phosphate (S1P) concentration by immunoassay in 3 affected subjects (F1-1, F1-2, and F2-1) and 4 unaffected controls revealed no difference between the groups (affected, 6.70 ± 1.14 [SD] μM; unaffected, 6.67 ± 1.64 μM).

### Effect of *SGMS2* pathogenic variants on enzyme localization and de novo sphingomyelin biosynthesis

**Figure 6:**
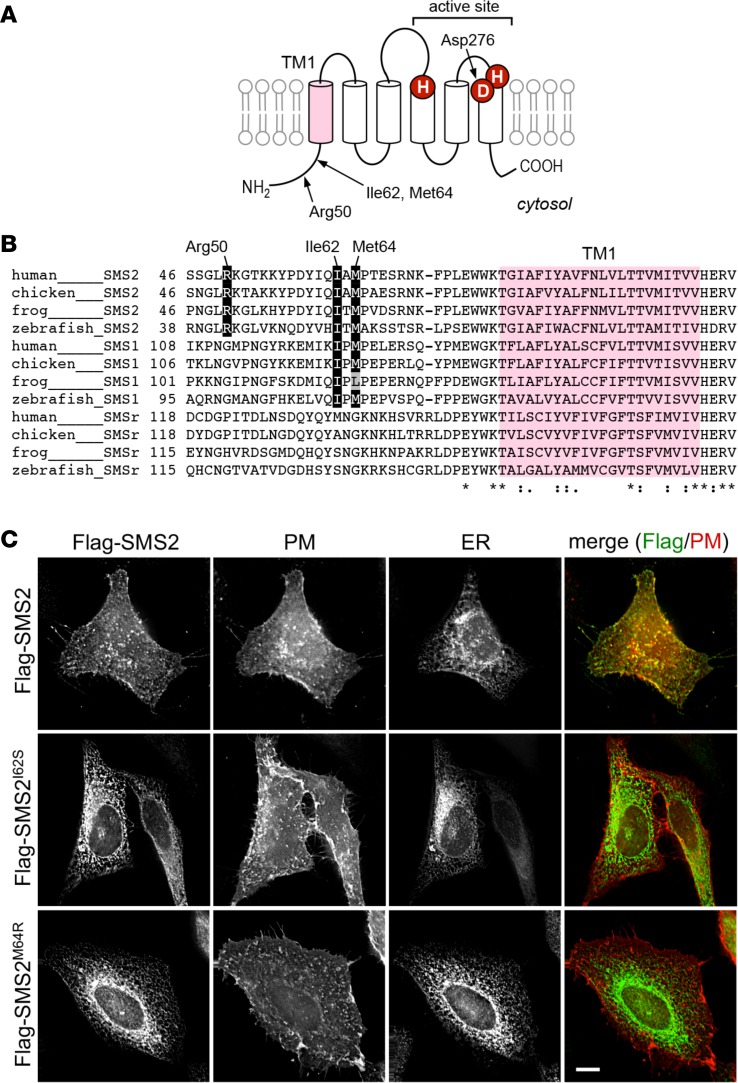
*SGMS2* missense variants p.Ile62Ser and p.Met64Arg block SMS2 export from the ER. (**A**) Predicted membrane topology of SMS2. Active site residues and the first of 6 membrane spans, TM1, are marked in red and purple, respectively. The positions of 3 residues substituted by osteoporosis-associated *SGMS2*pathogenic variants, i.e., Arg50 (p.Arg50*), Ile62 (p.Ile62Ser), and Met64 (p.Met64Arg), are indicated. (**B**) Sequence alignment of the region immediately upstream of TM1 in vertebrate SMS family members. Note that the human SMS2 residues Ile62 and Met64 are conserved between SMS2 and SMS1, but not SMSr, across different vertebrate species. Database accession numbers for the sequences are human SMS2, Q8NHU3; chicken SMS2, F1NLG9; frog SMS2, Q28DN3; zebrafish SMS2, B8A5Q0; human SMS1, Q86VZ5; chicken SMS1, Q7T3T4; frog SMS1, F6UTK5; zebrafish SMS1, A0JMN0; human SMSr, Q96LT4; chicken SMSr, F1NI82; frog SMSr, F6SHG1; zebrafish SMSr, A4QNV5. (**C**) HeLa cells cotransfected with Flag-tagged SMS2, SMS2^I62S^, or SMS2^M64R^ and PM marker Lyn-FRB-mCherry (red) were costained with anti-Flag (green) and anti-calnexin antibodies (ER) and visualized by fluorescence microscopy. Scale bar: 10 μm.

**Figure 7:**
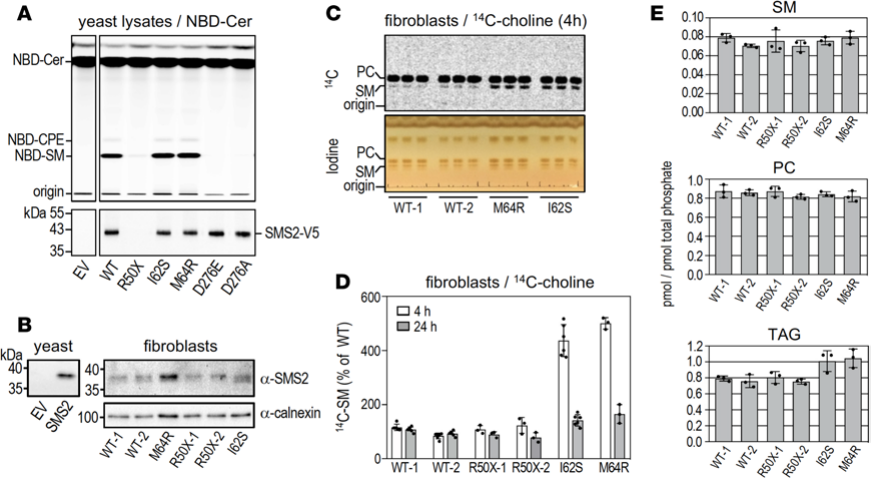
Effect of *SGMS2* pathogenic variants on SMS2 catalytic activity and sphingomyelin biosynthesis. (**A**) Top: TLC analysis of reaction products formed when lysates of yeast strains expressing WT or mutant versions of V5-tagged human SMS2 were incubated with C_6_-NBD-ceramide for 20 minutes at 37°C. Note that SMS2^I62S^ and SMS2^M64R^ produce similar amounts of NBD-sphingomyelin (NBD-SM) as WT-SMS2, whereas SMS2^R50X^ and the active site mutants SMS2^D276E^ and SMS2^D276A^ lack sphingomyelin synthase activity. EV denotes yeast lysate from strain transfected with empty vector. Bottom: SMS2 levels in yeast lysates were analyzed by immunoblotting using anti-V5 antibody. (**B**) Total membranes of control (WT) or patient-derived fibroblasts carrying heterozygous p.Arg50*, p.Ile62Ser, or p.Met64Arg nonsense and missense variants were subjected to immunoblot analysis using anti-SMS2 and anti-calnexin antibodies. Immunoblot of total membranes from yeast transfected with empty vector (EV) or SMS2-encoding plasmid was stained with anti-SMS2 antibody and served as control. (**C**) TLC analysis of lipid extracts from control (WT) or patient-derived fibroblasts carrying heterozygous p.Ile62Ser or p.Met64Arg missense variants grown for 4 hours in medium supplemented with ^14^C-choline. Incorporation of ^14^C-choline into phosphatidylcholine (PC) and sphingomyelin (SM) was analyzed by autoradiography (top). Lipids were stained with iodine vapor to verify that total lipid content between extracts was comparable (bottom). (**D**) Quantitation of ^14^C-choline incorporation into sphingomyelin in control or patient-derived fibroblasts treated as in **C**. Data are mean ± SD; *n* ≥ 3. (**E**) Sphingomyelin, phosphatidylcholine, and triacylglycerol (TAG) levels in total lipid extracts of control or patient-derived fibroblasts were determined by LC-MS/MS and expressed as mole percent of total phospholipid analyzed. Data are mean ± SD; *n* = 3.

The p.Arg50* mutation is predicted to yield a truncated SMS2 protein lacking the entire membrane-spanning core domain, which includes the active site (Figure 6). Heterologous expression of SMS2-Arg50* gave rise to a protein that was completely mislocalized in the cytosolic and nuclear compartments of HeLa cells (Supplemental Figure 11) and failed to support sphingomyelin production in yeast (Figure 7A). On the other hand, the p.Ile62Ser and p.Met64Arg missense variants did not interfere with SMS2 catalytic activity (Figure 7A). However, whereas WT SMS2 predominantly localized at the plasma membrane, both SMS2-Ile62Ser and SMS2-Met64Arg accumulated in the endoplasmic reticulum (ER) (Figure 6C). Ile62 and Met64 are part of a conserved sequence motif (I-X-M-P) located 13–14 residues upstream of the first membrane span in both SMS2 and the Golgi-resident isoenzyme SMS1 (Figure 6B). This motif may represent an ER export signal, in line with its absence in the ER-resident isoenzyme SMSr.

We next analyzed the consequences of *SGMS2* pathogenic variants on cell proliferation, endogenous SMS2 expression levels, and sphingomyelin biosynthetic capacity using fibroblasts derived from patients and healthy controls. We did not observe any variant-associated differences in cell proliferation, viability, or SMS2 transcript levels (data not shown). None of the variants had any major effect on cellular SMS2 protein levels (Figure 7B). SMS2 catalyzes a reaction in which the phosphocholine head group of phosphatidylcholine is cleaved off and transferred onto ceramide, yielding sphingomyelin and diacylglycerol (13). Metabolic labeling of patient-derived fibroblasts with ^14^C-choline indicated that the heterozygous p.Arg50* variant had no obvious effect on the cellular sphingomyelin biosynthetic capacity (Figure 7D and Supplemental Figure 12). However, in contrast to the p.Arg50* nonsense variant, heterozygous p.Ile62Ser and p.Met64Arg missense variants caused a marked increase in the rate of sphingomyelin biosynthesis (Figure 7, C and D). Moreover, while steady-state levels of sphingomyelin, phosphatidylcholine, ceramide, and diacylglycerol in patient-derived and control fibroblasts were largely comparable, fibroblasts carrying the p.Ile62Ser or p.Met64Arg missense variants contained elevated levels of triacylglycerol (Figure 7E and Supplemental Figure 13). As sphingomyelin biosynthesis is directly coupled to phosphatidylcholine turnover and the release of diacylglycerol, we postulate that an accumulation of diacylglycerol in the ER of p.Ile62Ser and p.Met64Arg fibroblasts is countered by its rapid metabolic conversion into triacylglycerol.

Collectively, our findings suggest that the underlying cause of osteoporosis and skeletal dysplasia in patients with pathogenic variants in *SGMS*2 is not a reduced cellular capacity to synthesize sphingomyelin but rather a perturbation in sphingomyelin metabolism at the plasma membrane.

## Discussion

We identified heterozygous pathogenic variants in the *SGMS2* gene encoding plasma membrane–resident SMS2 in patients with rare forms of osteoporosis. The clinical presentations ranged from isolated childhood-onset osteoporosis to severe generalized bone involvement (spondylometaphyseal dysplasia) with neonatal fractures and marked cranial hyperostosis. The milder phenotype was associated with the p.Arg50* nonsense variant, identified in 10 individuals in 4 unrelated families, whereas the more severe phenotype was associated with closely localized missense variants, p.Ile62Ser and p.Met64Arg. The p.Arg50* mutation resulted in expression of a catalytically nonfunctional enzyme, whereas the 2 missense variants caused retention of enzymatically active SMS2 in the ER, presumably by disrupting an ER export signal. All 3 pathogenic variants led to decreased bone mineralization in both cortical and trabecular bone. Our results demonstrate that SMS2 has bone-regulatory properties and point to a critical role of plasma membrane sphingomyelin metabolism in normal skeletal homeostasis.

Multiple factors contribute to bone strength, including bone mass, bone microarchitecture, and material properties related to bone matrix mineralization and arrangement of collagen and collagen fibrils (14, 15). Several of these characteristics were negatively affected by the *SGMS2* pathogenic variants. In particular, we observed a reduction in bone matrix mineralization. This seems to be due to alterations in the mineralization process itself rather than an abnormally high bone turnover (16, 17). This is in line with our finding that the bone resorptive capacity of patient-derived osteoclasts was unaffected. Moreover, bone biopsies showed that the regular lamellar bone matrix was severely disturbed (18). The mesh-like appearance of bone matrix and the marked increase in osteocyte lacunar density and size are characteristic for primary bone, known to be of low bone strength (19, 20). Moreover, microspectroscopy indicated deceleration of mineral accumulation kinetics with tissue maturation and low content of pyridinoline, which is needed for proper crosslinking of collagen fibrils. Although no biopsy material was obtained from the CDLs, one could speculate that they are caused by remnants of primary bone with an elevated degree of mineralization. The measured circulating bone turnover markers showed variable results and did not fully correlate with the bone tissue characteristics, which is in line with our previous observations that these markers reflect bone biopsy findings poorly in both children and adults (21, 22).

Inborn errors of phospholipid metabolism are emerging as relevant causes for disturbed bone homeostasis (23). In particular, alterations in the choline pathway have been documented in at least 2 skeletal dysplasia phenotypes. Pathogenic variants in the phosphatidylserine synthase 1 gene (*PTDSS1*) cause Lenz-Majewski syndrome (MIM #151050), characterized by craniofacial, dental, and limb abnormalities (24, 25). Pathogenic variants in the PCYT1A gene, encoding CTP-phosphocholine cytidylyltransferase, a key enzyme in the de novo phosphatidylcholine biosynthesis, cause spondylometaphyseal dysplasia with cone-rod dystrophy (MIM #608940) with normal development (26). Our study expands these findings by providing the first link to our knowledge between sphingolipid metabolism and a human bone disease. S1P, which is generated through ceramide deacylation followed by phosphorylation by sphingosine kinases, is known to play a fundamental role in bone metabolism, specifically in the functional coupling of osteoblasts and osteoclasts (27, 28). S1P lyase is emerging as a potential target for osteoporosis therapy (29). A defect in plasma membrane SMS2 activity could alter the local ceramide concentrations and disturb S1P synthesis. However, our analyses showed unaltered serum S1P concentrations in affected subjects, suggesting that the pathology is not S1P dependent.

**Figure 8:**
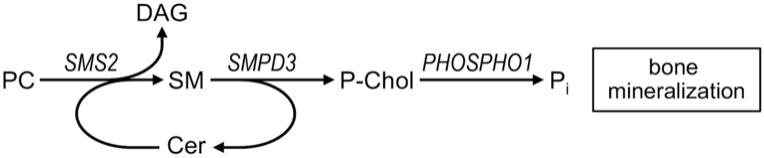
Model on the role of sphingomyelin metabolic enzymes in bone mineralization. Sphingomyelin phosphodiesterase 3, SMPD3, cleaves sphingomyelin (SM) in the exoplasmic leaflet of the plasma membrane to generate ceramide (Cer) and phosphocholine (P-Chol). P-Chol serves as a substrate for phosphocholine phosphatase PHOSPHO1 to release phosphate and promote bone mineralization. Plasma membrane–resident sphingomyelin synthase SMS2 regenerates SM from Cer released by SMPD3, using phosphatidylcholine (PC) as head group donor and forms diacylglycerol (DAG) as by-product. Coupling of SMPD3 and SMS2 enzymatic activities on the surface of osteoblasts enables a continuous supply of phosphocholine required for normal bone mineralization. Release of DAG, a potent signaling lipid, provides a potential cue for osteoblasts to fine-tune supply of lipid-derived phosphate.

In mice deletion of the gene encoding sphingomyelin phosphodiesterase 3 (Smpd3) impairs bone and cartilage mineralization, leading to severe skeletal deformities (30). Although a cell-autonomous role of Smpd3 in bone mineralization has been established (31), it remains unknown how Smpd3 contributes to normal skeletal homeostasis. Our present findings underscore a crucial role for sphingomyelin metabolism in skeletal mineralization. SMPD3 is a membrane-bound enzyme that cleaves sphingomyelin to generate ceramide and phosphocholine on the cell surface. Phosphocholine released by SMPD3 may provide a source of phosphate to promote bone mineralization (32–34). Unlike SMPD3, however, SMS2 catalyzes a reaction whereby phosphocholine is cleaved from phosphatidylcholine and subsequently transferred onto ceramide to generate sphingomyelin and diacylglycerol. Even though SMS2 can also catalyze the reverse reaction (13), the enzyme is unable to release phosphocholine and instead forms a histidine-phosphocholine intermediate during the reaction cycle (35). However, by regenerating sphingomyelin from ceramide released by SMPD3, SMS2 may serve to replenish the sphingomyelin pool used by SMPD3 to liberate phosphocholine during bone mineralization (Figure 8). The abundant expression of SMS2 in cortical bone implies a high demand for SMS2 activity at the plasma membrane to enable this process. All 3 *SGMS2* pathogenic variants identified in this study would diminish the pool of active enzyme at the plasma membrane by disrupting SMS2 catalytic activity (p.Arg50*) or ER export (p.Ile62Ser, p.Met64Arg). This may hamper a continuous supply of phosphocholine required for normal bone mineralization, causing osteoporosis.

Immunoblot analysis of patient-derived fibroblasts revealed that the p.Arg50* mutation has no major effect on total SMS2 expression, suggesting that expression from the unaffected allele may be increased to compensate for the loss of full-length protein from the affected allele. Whether this scenario also holds for patient-derived bone cells remains to be established. Given that SMS2 is abundantly expressed in cortical bone, further upregulation of SMS2 expression from the WT allele to compensate for the p.Arg50* mutation in bone cells may no longer be feasible. In addition, robust expression of a 49-residue-long *N*-terminal SMS2 fragment may have a dominant-negative effect on the bone regulatory properties of the full-length protein. As our anti-SMS2 antibody was raised against the protein’s *C*-terminus, we have not been able to verify the latter scenario.

A puzzling aspect is that removal of Sgms2 in mice ameliorates diet-induced obesity and insulin resistance but is not known to cause any overt bone defect (36, 37). While a bone phenotype in *Sgms2* KO mice may have been overlooked, the *SGMS2* pathogenic variants described in this study may also have a dominant-negative effect on bone formation. As SMS2 is known to form homodimers (38), expression of the p.Ile62Ser and p.Met64Arg missense variants may cause a partial retention of the WT enzyme in the ER. Bulk production of sphingomyelin in the ER may be detrimental for the central function of this organelle in calcium homeostasis, stress signaling, and secretion, in particular in bone cells where SMS2 is abundantly expressed. In fact, this may explain why the p.Ile62Ser and p.Met64Arg missense variants cause a more severe bone phenotype than the p.Arg50* nonsense variant. In addition, SMS2 serves as important crossover between 2 key signaling lipids with opposing biological effects: ceramide and diacylglycerol. Recent work revealed that SMS2-generated diacylglycerol at the plasma membrane is critical for the development of insulin resistance and controlled by GPRC5B-mediated SMS2 phosphorylation (39). Diacylglycerol is a key component of a PKCδ-operated pathway that promotes bone formation (40). Consequently, pathogenic variants in *SGMS2* may also affect normal skeletal homeostasis by perturbing diacylglycerol signaling at the osteoblast plasma membrane.

In addition to the skeletal disease, several subjects had neurological manifestations. The transient nerve palsies, most commonly involving the cranial nerve VII and presenting as Bell’s palsy, were more prevalent and had earlier onset in patients with missense variants as compared with patients with p.Arg50* mutation, who only had cranial nerve palsies at adult age. One patient with p.Ile62Ser was also diagnosed with progressive ataxia due to sensory neuronopathy. Recurrent facial nerve palsy has been reported in one patient with CDL (41), while several other publications on CDL (6, 42–44) lack information on potential neurological symptoms. There are several plausible mechanistic explanations: compression due to cranial sclerosis, as previously suggested (5); basilar invagination due to compromised bone strength, as in OI (45), although no evidence for that was noted in our patients; and neurotoxicity induced by aberrant sphingomyelin metabolism, similar to neuronal damage with incidental cranial nerve palsies in acid sphingomyelinase deficiency (Niemann-Pick disease) (46). In the patients with missense variants, accumulation of SMS2 and production of sphingomyelin in the ER could also lead to a lipid-mediated cellular stress response with adverse effects on neuronal function (47). The transient nature of the cranial nerve palsies remains unexplained. Further, it remains unclear whether the several other symptoms in our patients (Tables 1 and 2) are manifestations of *SGMS2* pathogenic variants.

In summary, our findings indicate that *SGMS2* pathogenic variants impair bone material properties, resulting in disturbances in mechanical competence; development and growth of the skeleton; and underlie a spectrum of skeletal conditions, ranging from early-onset osteoporosis to osteoporosis with skeletal dysplasia. These findings suggest a critical role for plasma membrane–bound sphingomyelin metabolism in skeletal homeostasis and in bone mineralization. Patients’ skeletal features, bone tissue characteristics, and the observed gene expression pattern, involving both osteoblasts and osteoclasts and being highest in the cortical bone, indicate a need to further explore the role of SGMS2 in osteoblast and osteocyte function. Moreover, recent studies (48, 49) have demonstrated expression of SGMS2 in primary chondrocytes isolated from patients with osteoarthritis, prompting the study of SGMS2 function also in this cell type. Our study underscores a wide range of presentations associated with *SGMS2* pathogenic variants and shows correlation with genotype. Bisphosphonate treatment seemed to have a beneficial effect, but long-term effects remain unknown. Further insights into the underlying molecular and biochemical mechanism may identify novel pharmacologic targets to improve bone strength.

## Methods

### Clinical studies

All study subjects were assessed as part of ongoing studies at the Helsinki University Hospital, the University of Utah, and the University Medical Center Utrecht. Patient data were collected by interview and from hospital records for fractures and other skeletal and nonskeletal morbidities, including neurological symptoms and evaluations. All patients were clinically examined.

### Genetic analyses

#### WES.

Genomic DNA was processed according to an Agilent protocol using the SureSelect Human All Exon v4 or v5 kit (Agilent Technologies Inc.) and sequencing with the Illumina HiSeq 2000 or 2500 (Illumina Inc.). Biological parentage was confirmed by short tandem repeat analysis. The reads were aligned to the hg19 reference genome, using the variant calling pipeline of the FIMM (50) or using the Genome Analysis Toolkit (GATK version 1.6, Broad Institute). The procedure yielded ×49 mean coverage of target bases. In family 1, reannotation was made with Annovar (51) and filtering with Excel and VarAFT 2.06 (http://varaft.eu). A comparison was made in family 1 between 2 affected subjects (the index and grandmother) and the healthy sibling to detect heterozygous variants shared by the 2 affected individuals but not present in the healthy sibling. Filtering of exome variants was done for rarity (variant frequency = 0.001 or less in public databases ESP6500, 1000 Genomes, ExAC, Kaviar, Haplotype Reference Consortium, SISu) and quality (WES) data with less than 15 reads per nucleotide. Seven variants were retained after this filtering procedure; these variants were Sanger sequenced in all family members for whom a DNA sample was available. Three variants were retained after Sanger sequencing: an Arg-to-stop gain change (c.148C>T/p.R50X) in the *SGMS2* gene coding for a sphingomyelin synthase 2, an Ile-to-Val change (c.A499G/p.I167V) in the *ALPK1* gene coding for alpha kinase 1, and an Asn-to-Val change (c.A335G/p.N112S) in the gene *ERC1* gene coding for ELKS/RAB6-interacting/CAST family member 1. After filtering variants for predicted deleteriousness (combined annotation dependent depletion [CADD] score = 25 or more), only the Arg-to-stop gain change (c.148C>T/p.R50X) in the *SGMS2* remained.

In families 3, 4, and 6 variants occurring in the known OMIM genes that may be associated with the phenotype and rare de novo and/or rare compound heterozygous variants occurring in genes of unknown clinical relevance were investigated. Common variants with a population frequency of greater than 0.001 were filtered out. The following population variant databases were used to aid variant filtering and interpretation: dbSNP 151, 1000 Genomes, gnomAD, and Mutation Database (HGMD) Professional 2017.4 (QIAGEN Bioinformatics).

In family 5, after referral for routine diagnostic exome sequencing, exomes of the affected patient (F5-1) and her healthy parents were enriched using the Agilent SureSelect XT Human All Exon kit V6 and sequenced in rapid 2 × 100 bp run mode on the HiSeq2500 sequencing system (Illumina Inc.). Reads were aligned to reference genome (hg19) using BWA-MEM algorithm (v.0.7.5a), and variants were called using the GATK haplotype caller (v3.4.46). Detected variants were annotated, filtered and prioritized using the Bench NGS Lab platform (Agilent-Cartagenia). Analysis is based upon a tiered analysis approach. The first tier that filters for de novo variants resulted in the detection of the de novo *SGMS2* variant. The last tier, which filters for recessive variants, did not result in additional candidates. The same heterozygous *SGMS2* variant was subsequently identified in the patient’s affected son (F5-2).

#### Whole-genome sequencing.

For patient F2-1 in family 2, we performed whole-genome sequencing on genomic DNA on the Illumina HiSeqX (Illumina Inc.) at the Science for Life Laboratory (NGI facility), Stockholm, Sweden. Parental samples were unavailable. Data alignment was performed with the BWA-MEM algorithm (0.7.12) to reference genome (hg19). GATK Best Practices recommendations (52–54) were followed to mark duplicates, recalibrate for base quality scores, realign indels, and call for variants using variant quality score recalibration (GATK, suite 3.3 and 3.6). Variants were annotated with the Variant Effect Predictor (version 86) and loaded as a database into GEMINI (0.19.0). Calls for known OI genes were then made with SAMtools (0.1.19) and BEDTools (2.23.0) to exclude pathogenic variants. Calls for variants in other new genes, including *SGMS2*, in which a mutation had previously been identified in family 1, were made. The computations were performed on resources provided by SNIC through Uppsala Multidisciplinary Center for Advanced Computational Science under project b2014258 (55).

#### Sanger sequencing.

We confirmed all detected *SGMS2* (NM_001136258) mutations, c.148C>T (p.R50X) (families 1–4), c.185T>G (p.I62S) (family 5), and c.191T>G (p.M64R) (family 6), with Sanger sequencing using standard protocols. In addition, we Sanger sequenced the *SGMS2* gene in 183 samples from healthy Finnish controls to exclude the presence of these variants. Sequencing was performed with an ABI 3730 DNA Analyzer (Applied Biosystems), and chromatograms were analyzed using Sequencher v5.0 software. Primer sequences and detailed PCR protocols are given in the Supplemental Methods.

### Imaging studies

We used radiography and dual-energy X-ray absorptiometry to characterize skeletal features. Radiological evaluations, including skeletal radiographs and magnetic resonance imaging and computed tomography evaluations of the brain and spine, were performed as part of the patients’ clinical care. Bone mineral density was measured by dual-energy X-ray absorptiometry and converted to *Z*-scores based on equipment-specific normative data adjusted for age and sex.

### Bone biopsy studies

We analyzed 2 transiliac bone biopsies, obtained from patient F1-1 and patient F2-1, as part of the diagnostic assessment of osteoporosis at age 12 and 15 years, respectively. The 2 transiliac bone biopsies were analyzed using histomorphometry, confocal laser scanning microscopy, polarized light microscopy, quantitative backscatter electron microscopy (qBEI), and Raman microspectroscopy to evaluate bone tissue characteristics. In addition, surgical bone biopsies from the femur were obtained from patient F5-1 at age 15 and 16 years; the bone samples were decalcified and embedded in paraffin. Sections were stained with H&E stain and examined by light microcopy for bone histology. All bone biopsies were analyzed simultaneously at the same center and with the same equipment.

Both transiliac bone biopsies were taken at Children’s Hospital, University of Helsinki, with a standard 7.5-mm trephine needle from anterior iliac crest (Rochester Bone Biopsy, Medical Innovations International). For tetracycline labeling, an identical protocol was used for each patient: 500 mg oral tetracycline was given 3 times a day in a 2:10:2-day sequence, as previously described (21). Patients also refrained from dairy products, calcium supplements, or antacids during the labeling. The transiliac bone biopsy samples were immediately fixed and stored in 70% ethanol and subsequently embedded in polymethylmethacrylate using standard procedures (21). Longitudinal sections of the bone sample cylinder were obtained. Three-μm-thick sections were obtained for bone histomorphometry. The surface of the residual sample block was flattened by grinding and polishing (Logitech PM5) so as to facilitate confocal laser scanning microscopy and qBEI.

The thin bone sections were stained with Goldner′s trichrome method (mineralized matrix green, unmineralized matrix/osteoid red). Using a light microscope (Axiophot, equipped with a digital camera, AxioCam HRc, Zeiss), structural parameters, as well as parameters of static bone formation and resorption were assessed. A confocal laser scanning microscope (Leica TCS_SP5, Leica Microsystems) was used to analyze parameters of dynamic bone formation from the sectioned bone area at the sample block surface. The histomorphometric outcomes were compared with age-appropriate reference values (56).

Bone matrix mineralization was characterized by measurement of the BMDD using qBEI (57). This was performed with a scanning electron microscope equipped with a 4-quadrant semiconductor backscatter electron detector (Zeiss Supra 40) after carbon coating of the sample surface. The entire cross-sectioned bone sample area was imaged with a spatial resolution of 3.6 μm/pixel. The gray levels, reflecting the mineral/calcium content, were calibrated by the material contrast of pure carbon and aluminum; thus, the resulting gray level histograms could be transformed into calcium weight percentage (wt% Ca) histograms as described previously (58). Five BMDD parameters were derived to characterize the BMDD (Supplemental Figure 5). The bone mineral density distribution BMDD outcomes of cancellous and cortical bone were compared with a young reference database (59).

The details of Raman microspectroscopy are presented in the Supplemental Methods.

### Gene expression studies

Gene expression in murine tissues, including cortical bone, and in in vitro–cultured primary murine calvarial osteoblasts, bone marrow–derived macrophages cultured in M-CSF, and osteoclasts differentiated from bone marrow macrophages in the presence of M-CSF and RANKL for three days was analyzed by real-time quantitative PCR. Details are given in the Supplemental Methods.

### Evaluation of osteoclast function

To obtain information on osteoclast function, we differentiated patients’ peripheral blood monocytes to osteoclasts and determined their characteristics and activity. In vitro osteoclast differentiation was done on plastic and bone. The details of these experiments are presented in the Supplemental Methods. Osteoclast bone resorption was evaluated by measuring the release of collagen type I fragments (CTX-I) in culture media using a commercial ELISA (IDS Immunodiagnostics Systems, catalog AC-07F1). Number of osteoclasts was counted in microscope or evaluated by measuring TRAP5b in culture media using a commercial ELISA (IDS Immunodiagnostics Systems, catalog SB-TR201A).

### Metabolomics and measurement of S1P

To investigate whether there are differences in blood metabolites relevant to bone homeostasis between the affected subjects and healthy controls, we investigated untargeted metabolomics using direct-infusion high-resolution mass spectrometry (Thermo Scientific) of dried bloodspots (Supplemental Methods). S1P concentration was measured with a Sphingosine 1 phosphate Elisa kit (Echelon Biosciences Inc.) from serum samples obtained from 3 affected and 4 unaffected subjects in family 1, according to the manufacturer’s instructions.

### Fibroblast cultures

The skin biopsy samples were put in sterile culture DMEM medium (Lonza, catalog BE12-614F), 20% FBS (Biowest, catalog S181B-500), 50 IU/ml penicillin, and 50 μg/ml streptomycin (Gibco). The skin biopsies were cut into small fragments in PBS on a Petri dish and transferred to Falcon tubes containing 3 ml PBS (without Mg and Ca) and 1000 U/ml Collagenase Type II: Clostridium histolyticum (Gibco). The tubes were incubated at +37°C for 2 hours. Collagenase inactivation was performed by addition of 3 ml ice-cold medium. The tubes were mixed by vortexing, and tissue lysates were centrifuged for 10 minutes, 150 *g* at +4 °C. The supernatants were removed, the cell pellets were resuspended in 900 μl DMEM and transferred to T-25 flasks (Corning Life Sciences). 4 ml of DMEM was added to each flask, and they were incubated in a 5% CO_2_ humidified incubator at 37°C for 1–2 weeks.

### Characterization of patient-derived and control skin fibroblasts

We analyzed cell proliferation, viability, *SGMS2* gene expression, steady-state sphingolipid content (ceramides, phosphatidylcholines, sphingomyelins), and the capacity of cells to synthesize sphingomyelin de novo in patient-derived and control skin fibroblasts.

To compare cell proliferation in cases versus controls, the same number of cells (5 × 10^3^) was cultured in a 96-well plate and the cells were counted at 30 hours or 1.5 × 10^5^ cells were cultured in the 6-well plates (3 wells for cases and 3 for controls) and the cells were counted at 24, 48, and 72 hours. Cell viability was measured at 30 hours with CellTiter-Glo 2.0 Assay (Promega).

RNA was isolated from patient-derived and control fibroblasts using the RNeasy kit (Qiagen), cDNA was synthesized with QuantiTect Reverse Transcription Kit (Qiagen), and gene expression analysis performed using *SGMS2* TaqMan Gene Expression assay (Hs00380453_m1) and for reference genes, *ACTB* and *TBP* TaqMan Gene Expression assays Hs99999903_m1 and Hs00427620_m1 (Applied Biosystems). qPCR reactions were run in CFX96 Real-Time systems (Bio-Rad). The data were normalized with amplification of *ACTB* and *TBP* genes. Threshold cycle values were determined using CFX Manager Software (Bio-Rad). Relative expression was calculated with the 2^ΔΔCt^ method (60, 61).

### Lipid composition of fibroblasts

To analyze the steady-state lipid composition of the fibroblasts, nearly confluent cells were washed twice with PBS and then incubated in DMEM containing 10% delipidated FCS for 72 hours in a cell incubator. After washing twice with PBS, cells were scraped from the dishes and the lipids were extracted (62). The extract was divided in two parts, one (30%) of which was used to analyze glycerophospholipids, while the other (70%) was subjected to alkaline hydrolysis to eliminate phosphatidylcholine, which would hamper quantification of sphingomyelin. The glycerophospholipid fraction was subjected to methylation, and the methylated glycerophospholipids were analyzed by liquid chromatography tandem–mass spectrometry (LC-MS/MS) using neutral loss scanning for detection (63). The instrument consisted of a Waters Acquity H-class UPLC and a Micro Premier triple quadrupole mass spectrometer (Waters Corporation). The column (Acquity BEH C4, 1 × 100 mm) was eluted with a gradient of 75% solvent A (ACN/water/5 mM NH4-formate) to 100 % solvent B (ACN/MeOH/5 mM NH4-formate) over 10 minutes. The sphingolipid fraction was likewise analyzed by LC-MS/MS, and sphingomyelin was detected by scanning for precursors of *m/z* 184, while other sphingolipids were detected by scanning for the precursors of *m/z* 264. The mass spectra were analyzed with the LIMSA software (64). Diacylglycerol and triacylglycerol were detected using multiple reaction monitoring and analyzed using the QuanLynx software (Waters Corporation). Quantification was based on the following internal standards: PC-28:2, PC-40:2, SM-15:0, SM-25:0, Cer-15:0, Cer-25:0, and DAG-40:2.

### Heterologous expression of SMS2 mutant proteins in HeLa cells

A cDNA insert encoding human SMS2 with a N-terminal FLAG tag (DYKDDDDK) was created by PCR and inserted into *Not*I and *Xba*I restriction sites of mammalian expression vector pcDNA3.1 (+). A yeast pYES2.1 expression construct carrying human SMS2 with a C-terminal V5 and polyHis-tag was described in Huitema et al. (13). Disease-related mutations in SMS2 were introduced using site-directed mutagenesis and verified by DNA sequencing. HeLa cells were cotransfected with pcDNA3.1-SMS2 constructs and the plasma membrane marker lyn-FRB-mCherry using Effectene (Qiagen). Cells were fixed 24 hours after transfection, immunostained with anti-FLAG and anti-calnexin antibodies, and then processed for fluorescence imaging as described previously (65).

### Catalytic activity of SMS2 mutant proteins

Yeast strain IAY11 (*MATa*, *ade2-1 trp1-1 can1-100 leu2-3,112 his3-11,15 ura3-52 ade3-*Δ*853*) was transformed with pYES2.1/SMS2-V5-His expression constructs and then grown in synthetic medium containing 2% (w/v) galactose to early mid-logarithmic phase. Cells were collected by centrifugation and washed in ice-cold buffer R (15 mM KCl, 5 mM NaCl, 20 mM HEPES/KOH, pH 7.2). The wet cell pellet (2 g) was resuspended in a final volume of 5 ml buffer R containing protease inhibitors (1 μg/ml aprotinin, 1 μg/ml leupeptin, 1 μg/ml pepstatin, 5 μg/ml antipain, 1 mM benzamidine, and 1 mM PMSF). Cells were lysed by vigorous vortexing with 3 g glass beads at 4°C with intermittent cooling on ice. A postnuclear supernatant was prepared by centrifugation at 700 *g* for 10 minutes at 4°C. After addition of 0.11 volume of glycerol, lysates were aliquoted, snap frozen in liquid nitrogen, and stored at –80°C. Lysates from various SMS2-V5–expressing yeast strains were normalized for SMS2-V5 levels and protein content by adding appropriate amounts of control lysate and then assayed for SMS activity with 25 μM C6-NBD-ceramide in a reaction volume of 100 μl. After 20 minutes at 37°C with constant shaking, reactions were stopped by addition of 3.75 volume CHCl_3_/MeOH (1:2 v/v) and subjected to total lipid extraction in protein LoBind tubes (Eppendorff) starting from a reference volume (1 volume) of 100 μl (sample) in 3.75 volume (375 μl) CHCl_3_/MeOH (1:2 v/v). After centrifugation at 21,000 *g* for 10 minutes at 4°C, the supernatant was transferred to a fresh tube containing 1 volume CHCl_3_ and 1.25 volume 0.45% NaCl in H_2_O. This 2-phase system was vortexed vigorously for 5 minutes and then centrifuged for 5 minutes at 21,000 *g* and 4°C to complete phase separation. The lower phase was carefully collected with a 1000 μl Hamilton syringe and transferred to a fresh tube containing 3.5 volumes of a 1:1 mixture of MeOH/0.45% NaCl in H_2_O and then vortexed and centrifuged as above. Finally, the lower phase was transferred to a fresh tube and dried in a Christ RVC 100-2-18 concentrator coupled to a Vacuubrand MZ 2C (9 mbar) diaphragm vacuum pump. The dried lipid film was dissolved in CHCl3/methanol (2:1), spotted at 120 nl/s on NANO-ADAMANT HP-TLC plates (Macherey-Nagel) using a CAMAG Linomat 5 TLC sampler, and then developed in a CAMAG ADC2 chamber with CHCl_3_/MeOH/25%NH_3_(aq) (50:25:6, v/v/v) as an eluent. NBD-fluorescence was detected using the Cy2 settings (470/535 Ex/Em) of a Typhoon FLA 9500 Biomolecular Imager (GE Healthcare).

### Statistics

Standard statistical methods were used for data analysis, as appropriate. Descriptive statistics are presented as mean ± SD. Results were considered significant at the 95% significance level (*P* < 0.05). In untargeted metabolomics, *Z*-scores were calculated based on average intensities and SD of the metabolites in controls, measured in the same technical runs. Student’s 2-tailed *t* test was used to compare *Z*-scores in affected patients versus unaffected family members. *P* values were adjusted using Bonferroni correction to control for false-positive results due to multiple testing.

### Study approval

All parts of the study were approved by institutional ethics committees (Helsinki University Hospital, University of Utah Institutional Review Board, and the Medical Ethical Committee of the University Medical Centre Utrecht), and informed consent was obtained from study participants and/or their legal guardians prior to the study. Subjects in families F-1 and F-2 were assessed as part of an ongoing research program at Helsinki University Hospital (HUS 1088/2016). In families F-3, F-4, and F-6, written consent was obtained for all evaluated family members for participation in the research project, including documentation of phenotypic data and the WES, through the process established for the Undiagnosed Disease Program and the University Phenotype Core and approved by the University of Utah Institutional Review Board. In family, F-5 routine diagnostic exome sequencing was performed with written consent of the patients and their parents. The consent for diagnostic WES was approved by the Medical Ethical Committee of the University Medical Centre Utrecht. Later, written consent was obtained for publication of medical history and outcome of WES analysis.

## Author contributions

MP, PAT, LDB, PH, MK, MAK, JJ, PS, VMO, KK, JCMH, and OM designed research studies. MP, PAT, LDB, PH, REM, PR, AJ, MK, MAK, EPP, KVG, MM, PBT, MKM, JJ, MK, JCC, PS, UHL, VMO, KK, JCMH, and OM collected data. MP, PAT, LDB, PH, REM, PR, AJ, MK, MAK, MKM, MK, JCC, PS, UHL, VMO, JCMH, and OM analyzed data. MP, PH, AJ, MK, MAK, MKM, MK, PS, UHL, VMS, and JCMH performed cell experiments. PR, EPP, and KK analyzed bone biopsies. MP, PTA, LDB, PH, REM, PR, AJ, MK, MAK, EPP, KVG, MM, PBT, MKM, JJ, MK, JCC, PS, UHL, VMS, KK, JCMH, and OM interpreted the data. MP, PAT, LDB, PH, REM, PR, AJ, MK, MAK, EPP, KVG, MM, PBT, MKM, JJ, MK, JCC, PS, UHL, VMO, KK, JCMH, and OM drafted the manuscript. MP, PAT, LDB, PH, REM, PR, AJ, MK, MAK, EPP, KVG, MM, PBT, MKM, JJ, MK, JCC, PS, UHL, VMO, KK, JCMH, and OM approved the final version of manuscript.

## Supplementary Material

Supplemental data

## Figures and Tables

**Table 2:**
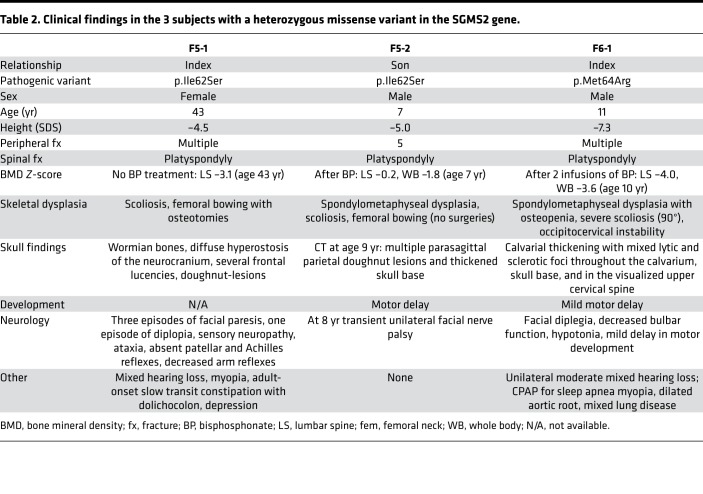
Clinical findings in the 3 subjects with a heterozygous missense variant in the SGMS2 gene.

**Table 1:**
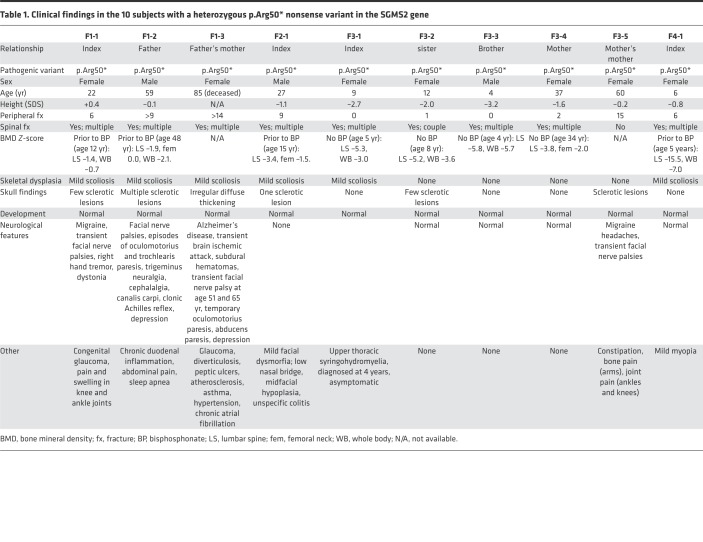
Clinical findings in the 10 subjects with a heterozygous p.Arg50* nonsense variant in the SGMS2 gene

## Version 1 02/19/2019

In-Press Preview

## Version 2 04/04/2019

Electronic publication
