# Supplementary material for: Osteoporosis and skeletal dysplasia caused by pathogenic variants in SGMS2
Source: JCI Insight. 2019 Apr 4;4(7):e126180. doi: 10.1172/jci.insight.126180 (PMC6483641; doi:10.1172/jci.insight.126180)
Supplement: Supplemental data [file jciinsight-4-126180-s209.pdf]

Supplemental Methods, Data, Figures and Tables (Pekkinen, Terhal, Botto, et al.)

1. SUPPLEMENTARY MATERIALS AND METHODS

PCR AMPLIFICATION OF THE *SGMS2* GENE

PCR amplifications were conducted in 20 µl containing 20 ng of DNA, 10 µl DreamTaq master mix (Thermofisher, Thermo Scientific™, Bremen, Germany) and 0.25 µM of each primer. Primers used for PCR reactions were: exon 1 (forward primer 5'- GCTAAATTTCCACAAAGAACAAGAA -'3 and reverse primer 5'- TCTCAATAACCCATCTATTCACTCA -3'); exon 2 (forward primer 5'- TTTCATTAGGAGTAAAACCAACCA -'3 and reverse primer 5'- CACATAGGGAAGTGTCTAGAAGAATG -3'); exon 3 (forward primer 5'- TCACAACCTGGTGTCCTTTCT -'3 and reverse primer 5'- GGGCTCCTATCAATTTCAGG -3'); exon 4 (forward primer 5'- GGTCGTTAGGACAGACTGTAATCAT -'3 and reverse primer 5'- ATCGGTTATCCCCATTAGGC -3') and exon 5 (forward primer 5'- TCCCTAGGTTACAGTGAATGACAA -'3 and reverse primer 5'- TCATTTACCAGTCAGTTCTCCTAAAA -3'). The amplification protocol was run on a Biorad DNA Engine Tetrad2 (Peltier Thermal Cycler, Mexico). After denaturation at 95 °C for 3 min, the reactions underwent 39 cycles of: 30 s at 95 °C, annealing for 30 s at 55–60 °C, and extension for 1 min at 72 °C. This was followed by a single step at 72 °C for 15 min.

SKELETAL CHARACTERIZATION

Raman microspectroscopy

We employed a Senterra (Bruker Optik GmbH, Ettlingen, Germany) instrument for Raman microspectroscopy. A continuous laser beam was focused onto the sample through a Raman fluorescence microscope (Olympus BX51, objective 50x, Olympus, Tokyo, Japan) with an excitation of 785 nm (100 mW) and a lateral resolution of ~0.6 µm (1). The Raman spectra were acquired from the biopsy block polished surface, using a thermo-electric–cooled charge-coupled device (Bruker Optik GmbH, Ettlingen, Germany). All data analyses were done with the Opus Ident software package (OPUS 6.5, Bruker Optik GmbH,

Ettlingen, Germany). Once acquired, the Raman spectra were baseline corrected (rubber band, 5 iterations) to account for fluorescence, and the following previously described (1). Raman parameters were calculated:

i. The mineral/matrix ratio was expressed as the ratio of the integrated areas of the v2PO4 (410–460 cm−1) to the amide III (1215–1300 cm−1) bands. This parameter reports the amount of mineral per amount of organic matrix per volume analyzed and has been shown to correlate with ash weight measurements (2) and to be directly proportional to bending stiffness and failure moment (3).

ii. Nanoporosity (approximated by the ratio of the integrated areas of the spectral slice 494-509 cm-1 of polymethylmethacrylate (PMMA) and Amide III band, a recently introduced parameter (4) that was not considered in our previous work (5) served as a proxy for tissue water content. In bone tissue, water generally exists within pores and bound to the matrix (6). Within the voxel analyzed (in our case 1x1x1.5 μm^3), PMMA will occupy space that is void of either mineral or organic matrix, thus in the present case the pores containing PMMA are in the sub-micron scale (hence the term "nanoporosity"). Matrix-bound water is not a monolayer but rather a series of coordinated water molecules, the outermost of which may be removed by the tissue processing (dehydration through a series of alcohols, acetone, and subsequent PMMA embedding). Thus, the water content inferred in the present study may be loosely coordinated water as well as water that was present in canaliculi. Bone tissue water content has been shown to influence the mechanical properties of bone (6) and was recently found altered in a mouse model for osteogenesis imperfecta (7).

iii. The relative glycosaminoglycan (GAG) content was defined as the GAG / total organic matrix ratio, calculated from the ratio of the integrated areas of the CH3 band ~ 1365–1390 cm−1 (characteristic of GAGs) (8) to the amide III bands (1215–1300 cm−1), respectively. Previously, this band was identified as proteoglycans (5) which are protein backbones decorated with GAGs. Since we actually use the CH3 band from the GAG chain, we use here the more precise denomination, GAG.

iv. Finally, the relative pyridinoline content (a major trivalent collagen cross-link) was calculated as the absorbance height at 1660 cm-1/area of the amide I (1620 – 1700 cm-1) (2,9). Enzymatic collagen cross-links have been shown to affect both stiffness and toughness (10).

EVALUATION OF *SGMS*2 GENE EXPRESSION

The mRNA expression of *Sgms2* in different tissues was evaluated in 22-weeks old female C57BL/6N mice (Charles River). Mice where anesthesized with Ketador/Dexdomitor (Richter Pharma/Orion Pharma), bled, and euthanized by cervical dislocation. Soft tissues were dissected, snap-frozen in liquid nitrogen, and stored at -80C until RNA preparation. Flushed mid-diaphysel tibial bone (cortical bone) and vertebral body L6 were stored in RNAlater at -80C until RNA preparation.

Liver, kidney, spleen, lung, muscle, heart, aorta and thymus were homogenized in RLT buffer with 1% 2-mercaptoethanol using a TissueLyser, followed by RNA preparation using the RNeasy mini kit (Qiagen). Bone tissues (cortical bone and vertebra), fat (retroperitoneal, gonadal and brow fat), brain cortex and hypothalamus were homogenized in TRIzol reagent (Life Technologies, Carlsbad, CA, USA) using a Tissue Lyser. After centrifugation to remove cell debris, the TRIzol homogenate was mixed with an equal volume of chloroform, centrifuged and the aqueous phase recovered. The aqueous phase was mixed with an equal volume of 70% ethanol and added to an RNeasy spin column. Thereafter the isolation followed the protocol from the RNeasy mini Kit (Qiagen).

Murine bone marrow macrophages and osteoclasts were cultured from bone marrow from male C57BL/6 mice as previously described (11,12). Bone marrow macrophages were cultured in 30 ng/ml M-CSF or in 30 ng/ml M-CSF and 4 ng/ml RANKL for 72 h to induce osteoclast differentiation. Cells were lysed in RLT buffer with 1% 2-mercaptoethanol, RNA was isolated using the RNeasy micro kit (Qiagen).

Primary osteoblasts were isolated by sequential digestion of calvarial bone dissected from 3-5 days old C57Bl/6N pups as previously described (13,14). Primary osteoblasts were cultured in the absence or

presence of 100 ng/ml BMP-2 for 7 days prior to lysis in RLT buffer with 1% 2-mercaptoethanol, and RNA isolation using the RNeasy micro kit (Qiagen).

RNA was converted to cDNA using High-Capacity cDNA Reverse Transcription kit (Applied Biosystems). Quantitative real-time PCR analysis for *Sgms2* was performed in the StepOnePlus Real-Time PCR system using pre-designed TaqMan Assay (Assay no Mm00512327_m1) and 18S as internal control (cat no 4310893E).

EVALUATION OF OSTEOCLAST FUNCTION

FACS analysis of peripheral blood monocytes

Peripheral blood monocytes can be divided into subtypes based on their surface expression of CD14 and CD16: classical ($CD14^{+}CD16^{-}$), non-classical ($CD14^{low}CD16^{+}$) and intermediate ($CD14^{+}CD16^{+}$) monocytes. The classical $CD14^{+}CD16^{-}$ monocytes are considered the major pool of osteoclast progenitors in peripheral blood. We analyzed different monocyte subpopulations by fluorescence-activated cell sorting (FACS). Peripheral blood mononuclear cells (PBMC) were isolated by Ficoll-Paque PLUS gradient centrifugation of blood collected in Li-Heparin tubes following the manufacturer's instruction. PMBC:s were stained for surface expression of CD14 (anti-CD14-APC, BD Pharmingen 555399, dilution 1:20), CD16 (anti-CD16-FITC, BD Biosciences 335035, dilution 1:20), CD51/CD61 (anti-CD51/CD61-PE, BD Pharmingen 550037, dilution 1:50) and M-CSFR (anti-M-CSFR-PE, AbCam ab95731, dilution 1:50) in blocking solution (0.3% AB-serum, 0.7% mouse serum and 1% fetal calf serum in 0.17 mM EDTA in PBS). Flow cytometry was performed using a BD FACSCanto II cytometer (BD Biosciences, San Jose, CA) and the results were analyzed using Flow Jo software.

In vitro osteoclast differentiation on plastic and bone

Two separate *in vitro* osteoclast differentiation experiments were performed. In the first experiment, monocytes from F1-1 and two age and gender matched controls and F2-1 and one age and gender matched

control were differentiated into osteoclasts on plastic and bone. In the second experiment, more age and gender matched healthy controls were included and the resorptive capacity analyzed. For all *in vitro* osteoclast differentiation experiments, $CD14^+$ monocytes were purified from PBMCs using CD14 MicroBeads and MACS columns according to the manufacturer's instructions (Miltenyi Biotec GmbH, Bergisch Gladbach, Germany). Cells were then seeded in 96-well plates ($3x10^5$ cells/cm^2) with or without devitalized bovine bone discs (IDS Immunodiagnostics Systems cat no TDT-1BON1000-96). Three to four wells per treatment condition were made. Cells were cultured in complete α-MEM medium (Gibco cat no 22561-021) supplemented with 10% heat inactivated fetal bovine serum (FBS, Sigma cat no F7524), 2 mM GlutaMAX (Gibco cat no 35050-038), 50 µg/ml gentamicin (Gibco cat no 15750-037), 100 U/ml penicillin and 100 µg/ml streptomycin (Gibco cat no 15140-148), 30 ng/ml human M-CSF (R&D Systems cat no 216-MC-025/CF) and 2 ng/ml recombinant mouse RANKL (R&D Systems cat no 462-TEC-010). Media was replenished every three or four days and the cells were stained for tartrate-resistant acid phosphatase (TRAP) at different days of culture using the Acid Phosphatase, Leukocyte (TRAP) Kit from Sigma (cat no 387A). $TRAP^+$ cells containing three or more nuclei were counted as $TRAP^+$ multinucleated osteoclast (MuOCL). The average number of osteoclasts per well or bone disc was calculated from 3-4 wells/bone discs.

Analysis of in vitro osteoclast bone resorption

$CD14^+$ monocytes were cultured on bone discs as described above for 14 days. Bone resorption pits on bone discs were visualized by reflective light microscopy. The amount of collagen type I fragments (CTX-I) released was measured in the cell culture media at the time of media renewal (every three to four days), using a commercial ELISA (IDS Immunodiagnostics Systems cat no AC-07F1). TRAP5b was analyzed in culture media using commercial ELISA (IDS Immunodiagnostics Systems cat no SB-TR201A).

Staining for the actin ring

Actin organization was studied at day 8 on bone. Cells were fixed in 4% paraformaldehyde, washed with PBS and then permeabilized using 0.1% Triton X-100 in PBS for 10 min at 4°C. After washing again with PBS, the cells were stained with rhodamine conjugated phalloidin (Life Technologies cat no R415), 5 U/ml in 2 % BSA/PBS, for 20 min at 4°C. Following washing in PBS the cells were mounted in Prolong Gold Mountant (Life Technologies cat no P-36931).

METABOLOMICS

We performed untargeted metabolomic studies with Direct-infusion High-resolution mass spectrometry from dried whole blood spots of 12 patients, 8 healthy family members and in-house controls. Dried blood spot sample extraction (3 mm, ~3.1 µL whole blood) was performed by addition of 140 µL IS methanol, followed by a 20-minute ultra-sonication step. Samples were diluted with 60 µL 0.3% formic acid and filtered using a methanol preconditioned 96 well filter plate (Acro prep, 0.2 µm GHP, NTRL, 1 mL well; Pall Corporation, Ann Arbor, MI) and a vacuum manifold. The filtrate was collected in a 96 well plate (Advion, Ithaca, NY). A TriVersa NanoMate system (Advion, Ithaca, NY) controlled by Chipsoft software (version 8.3.3, Advion) was mounted onto the interface of a Q-Exactive high-resolution mass spectrometer (Thermo Scientific™, Bremen, Germany). The Q-Exactive high-resolution mass spectrometer was operated in positive and negative ion mode with automatic polarity switching. Scan range was 70 to 600 m/z, resolution was 140,000 at m/z = 200. Samples were analyzed in triplicate.

Data acquisition was performed using Xcalibur software (version 3.0, Thermo Scientific™, Bremen, Germany). Using MSConvert15 (ProteoWizard Software Foundation), raw data files containing scanning time, mass over charge (m/z) and peak intensity were converted to mzXML format in Profile mode. A peak calling pipeline was developed in R programming language. Mass peak identification and annotation was conducted by matching the m/z value of the mass peak with a range of two parts per million to metabolite masses present in the Human Metabolome Database, version 3.6 (15). Z-scores for each metabolite were calculated based on control samples measured in the same run and the metabolites were ranked based on Z-scores; a Z-score of 1 meaning the metabolite is elevated with one standard deviation compared to in-

house controls (n=20-40). A metabolite level was regarded elevated when the Z score was >1 and decreased when the Z score was <-0.8 when compared to in-house controls. In addition, metabolites of interest were excluded if they were altered in unaffected controls (family members) provided by the same center as the patients as such alterations are likely due to artefacts (differences in type of Guthrie card or storage conditions). Multiple comparisons t-test with Bonferroni correction was used.

2. SUPPLEMENTAL FIGURES

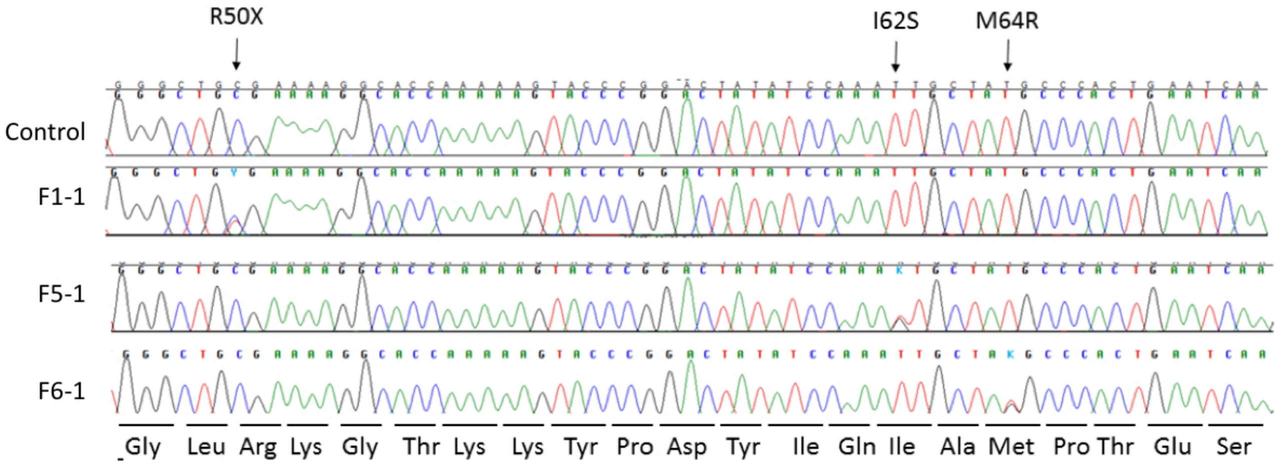

Figure S1. Sanger sequences of the identified SGMS2 mutations. The nucleotide sequences of *SGMS2* in a control and three affected subjects. Patient F1-1 is heterozygous for a single nucleotide change c.148C>T which generates a stop codon at position 50 (Arg50*). Patient F5-1 is heterozygous for a single nucleotide change c.185T>G leading to an amino acid change from isoleucine to serine (Ile62Ser). Patient F6-1 is heterozygous for a single nucleotide change c.191T>G leading to an amino acid change from methionine to arginine at position 64 (Met64Arg).

Figure S2. Additional radiographs of the patients with *SGMS2* mutations.

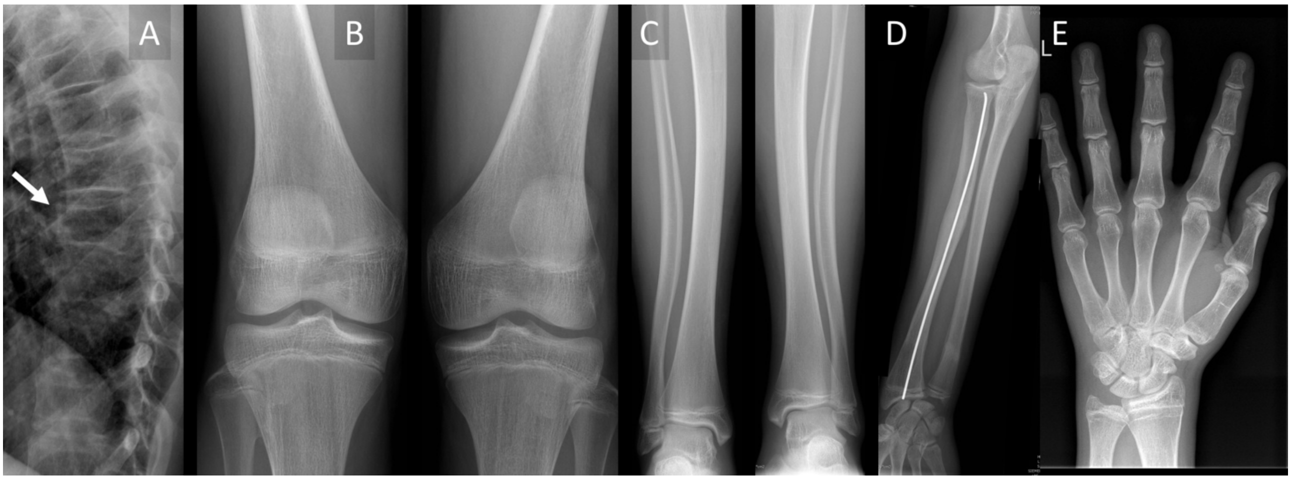

Radiographs of Patient F2-1 with the p.Arg50* mutation at the age of 15-17 years showing a vertebral compression fracture in the thoracic spine (arrow) (A), mild bowing of femurs and osteopathia striata-looking bone texture (B), bowing of fibulae (C), osteopenia and an intramedullary rod after forearm fractures (D), and hand radiograph with mild undertubulation of the long bones.

Figure S3. Additional radiographs of the patients with *SGMS2* mutations.

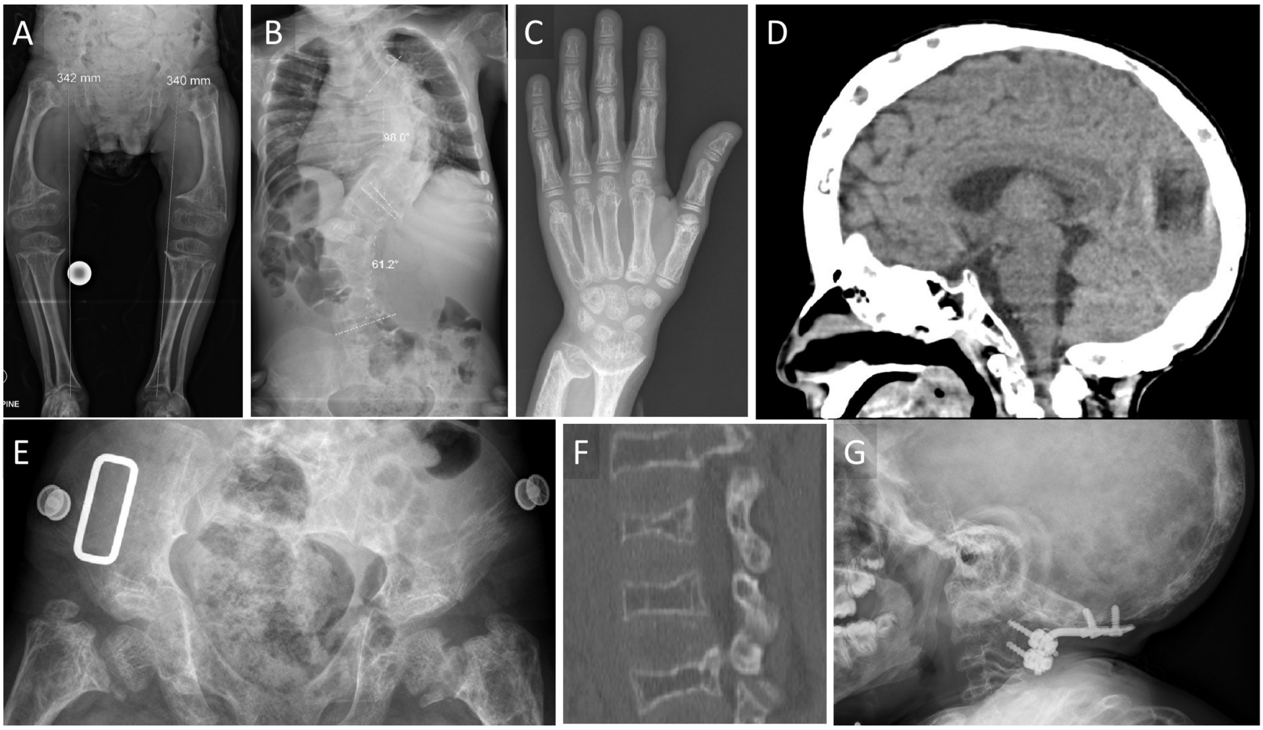

Radiographs of patient F6-1 with the p.Met64Arg mutation. At 11 years he has significant short stature with short and wide long bones, especially femurs, with metaphyseal widening and abnormal bone structure (A) and severe scoliosis (B). Metaphyseal changes and abnormal sclerotic bone structure can also be seen in his hand and wrist (C). Computerized tomography of the skull shows severe calvarial thickening with mixed lytic and sclerotic foci throughout the calvarium, skull base, and visualized upper cervical spine (D). Pelvis and proximal hips show sclerotic, clearly abnormal bone structure with coxa vara and abnormal metaphyses in the proximal femurs (E). Spinal abnormalities with poor mineralization and platyspondyly are seen on computerized tomography already at 3 years (F). The patient has undergone cervical occipital fusion; cervical vertebrae are small and osteopenic and the skull bone is sclerotic and irregular in texture (G).

Figure S4. Bone histology in a femur biopsy.

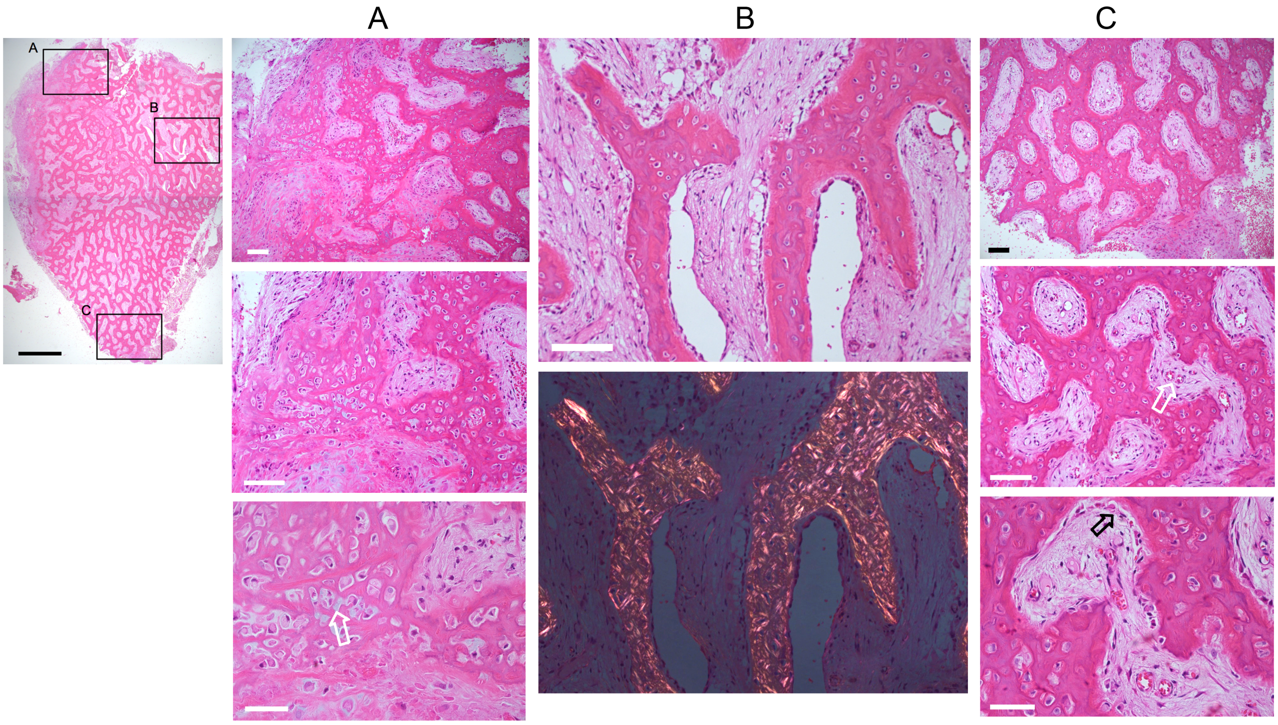

A, B and C: De novo bone formation out of a fibrous tissue environment. Details of region A: column of images with increasing magnification (scale bars from up to down 100 µm, 100 µm and 50 µm) shows features of a more likely cartilage matrix with chondrocytes (chondrons, white arrow) than bone matrix with osteocytes. Details of region B: Seams of cells (most likely osteoblasts) on the trabecular surface can be seen (upper image, scale bar 100 µm). In polarized light (bottom image) the haphazard arrangement of the collagen fibrils is visible indicating the presence of woven bone. Details of region C: column of images with increasing magnification (scale bars from up to down 100 µm, 100 µm and 50 µm) represents trabecular features in a mixture of bone and cartilage like tissue. The space between the trabeculae is filled with fibrous tissue matrix produced by fibroblasts. Numerous blood vessels (white arrow) are embedded in it. Osteoblast-like cells forming a seam on trabecular surfaces (black arrow) can be observed.

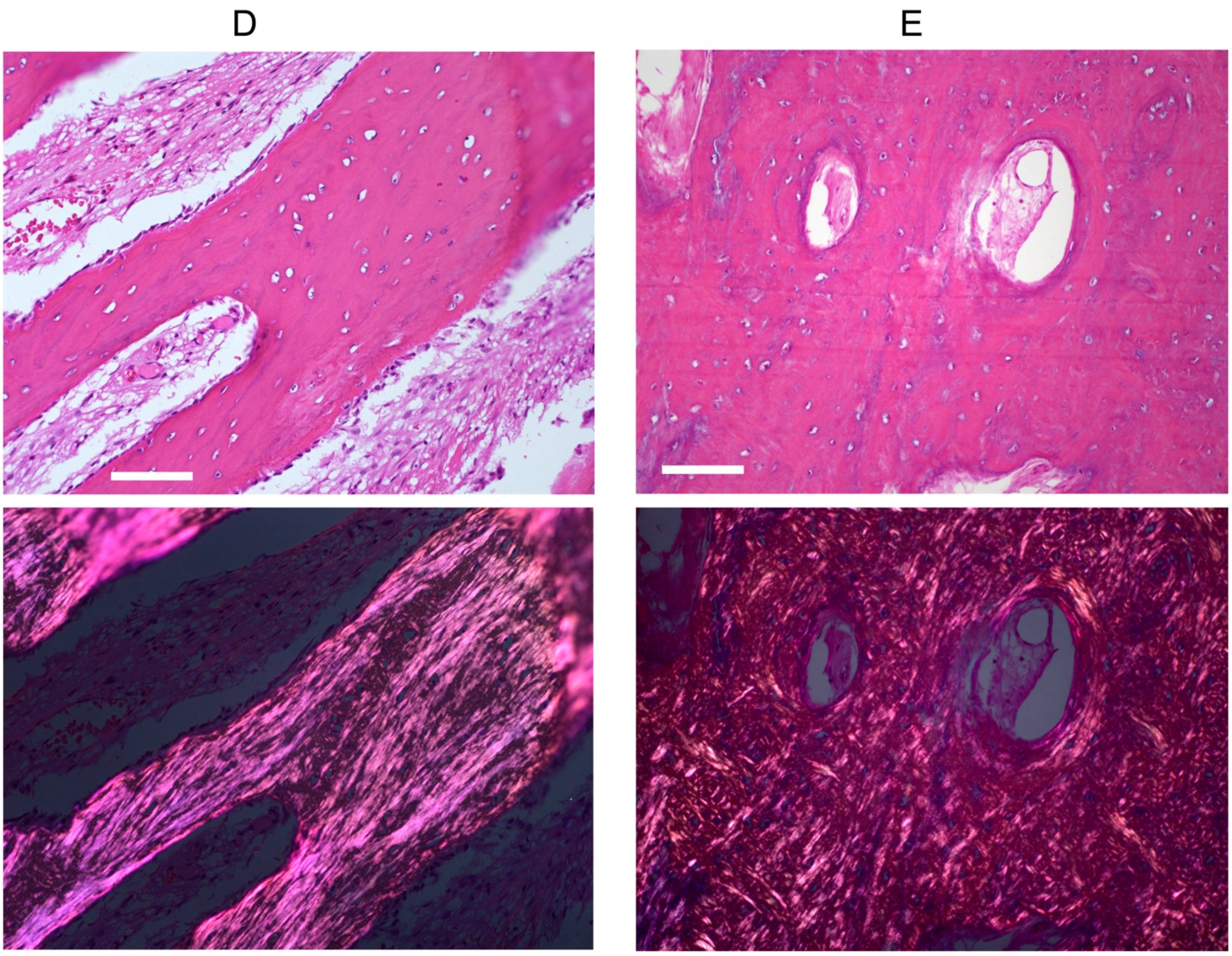

D: Bone fragment with more compact trabecular features. The trabecular bone matrix contains exclusively osteocytes/lacunae (E top, scale bar 100 µm). The collagen fibrils are partly orientated parallel to the trabecular surfaces (E bottom), but there is no lamellar arrangement like in mature bone. The bone marrow space is filled with fibrous matrix.

E: Bone fragment of a more compact cortical bone. Bone matrix with numerous osteocytic lacunae and larger voids/canals, most likely primary osteons (E top, scale bar 100 µm). In polarized light (E bottom) a clear haphazard arrangement of the collagen fibrils is visible (woven bone). A lamellar arrangement of collagen fibrils (secondary osteons) is lacking.

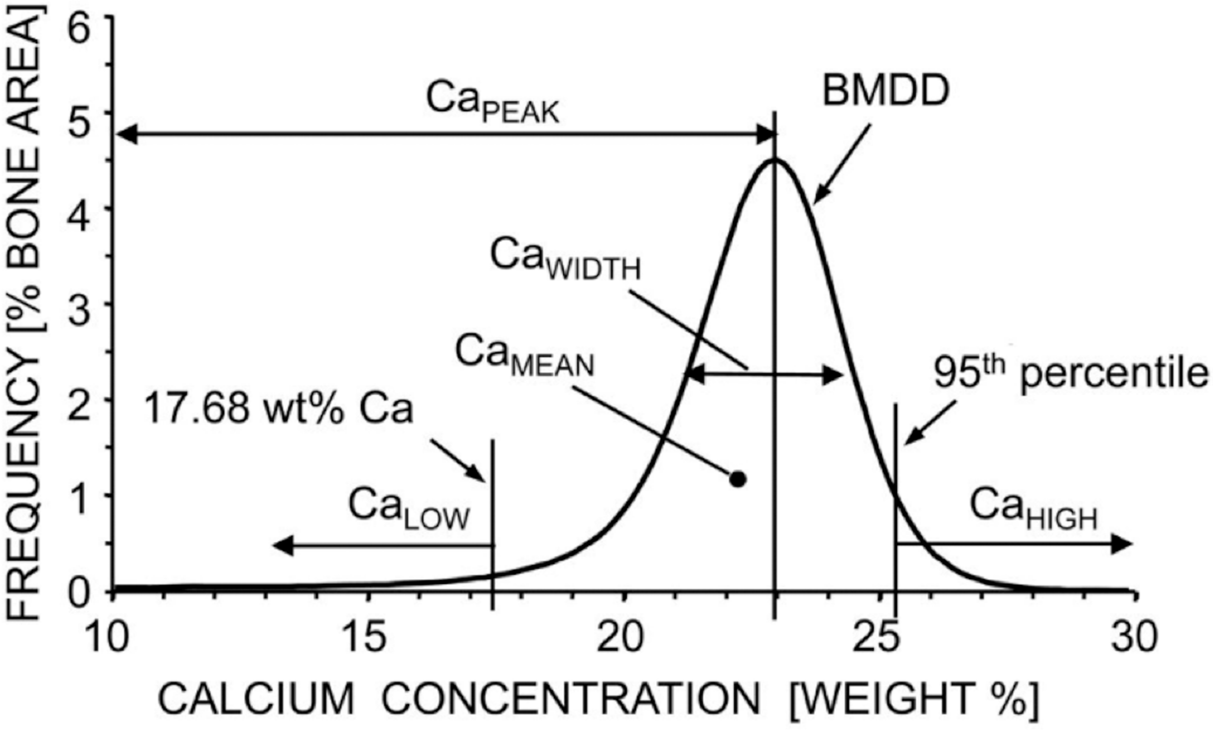

Figure S5. **The five BMDD parameters in quantitative backscatter electron microscopy**. CaMean: the mean calcium concentration (weighted mean); CaPeak: the most frequently occurring calcium concentration in the sample (the peak position of the BMDD); CaWidth: the width of the BMDD distribution (full width at half maximum) reflecting the heterogeneity in matrix mineralization; CaLow: the percentage of low mineralized bone area, which is mineralized below 17.68 wt% Calcium reflecting normally bone areas undergoing primary mineralization; CaHigh: the percentage of highly/fully mineralized bone matrix, which has a calcium content above 25.30 wt% Ca (corresponding mainly to interstitial bone).

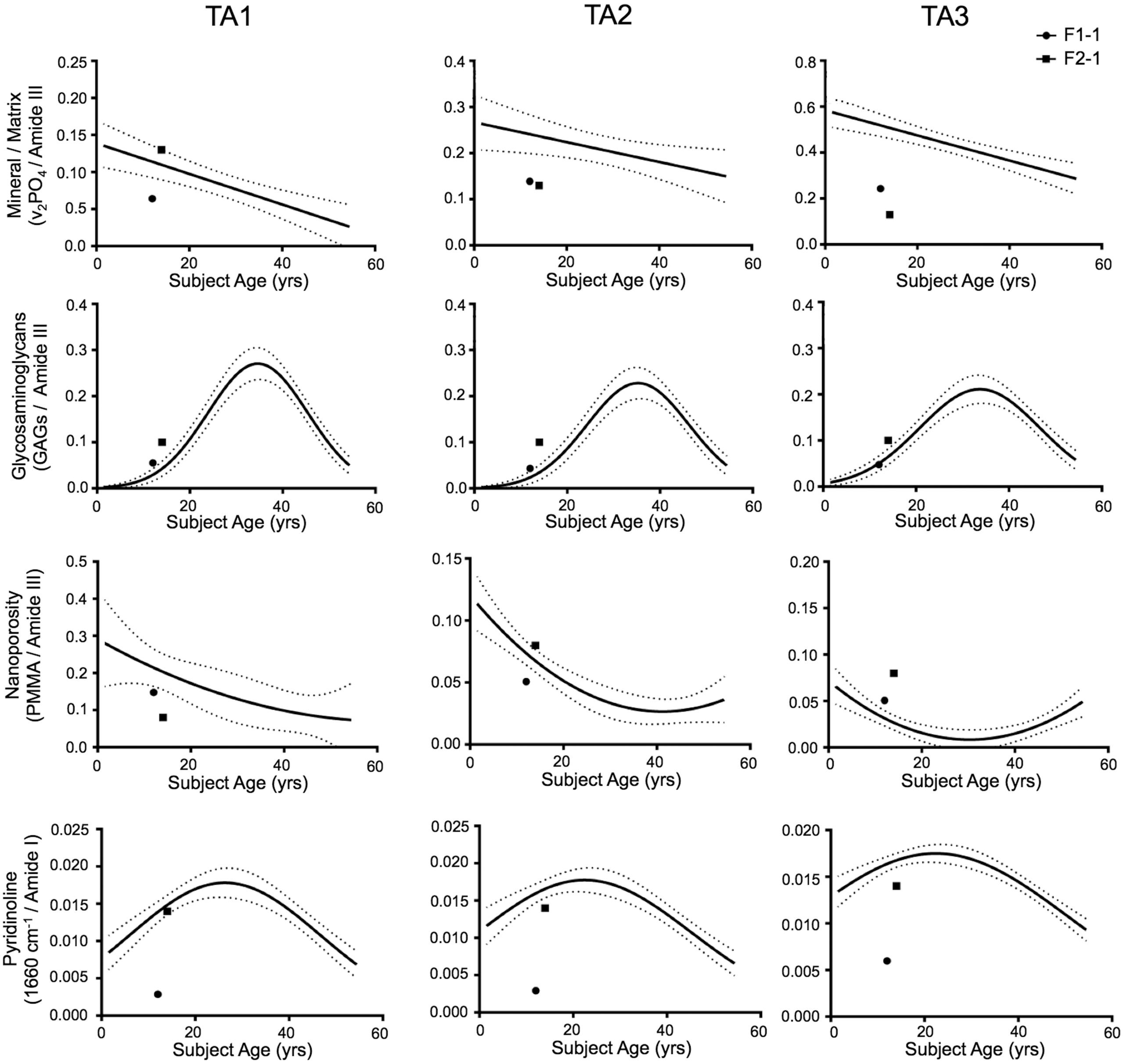

Figure S6. Findings in Raman spectroscopy analysis of transiliac bone biopsies. Transiliac bone biopsies were obtained from patients F1-1 and F2-1. F1-1 had fluorescent labels unlike F2-1. Therefore, area selection for analysis was based on the double labels for F1-1 (precise tissue age) while for F2-1 it was based on Ca content in qBEI images (selected areas with low Ca content; ongoing bone formation). Outcome values were compared with previously published regression models describing the variation of the spectroscopic parameters in healthy females as a function of chronological age as well as tissue age within the same donor (16). Three tissue ages were analyzed; in F1-1, TA1 = mid-distance between the

second label and the mineralizing front, TA2 = mid-distance between the two labels, TA3 = 2 μm behind the first label. In F2-1, distances were matched from the mineralizing front in qBEI. The results indicate that:

1. Mineral/matrix: At TA1, there were no striking differences between the 2 cases and age-matched healthy. But both patients had lower values at TA2 and TA3, suggesting a deceleration of mineral accumulation kinetics.

2. Nanoporosity (a surrogate for tissue water content) did not show any consistent trend. At TA3, the higher values would be consistent with the lower mineral/matrix ratio as mineral mainly displaces water in the bone tissue.

3. GAG content: In F1-1, values did not differ from healthy females, but F2-1 had higher values. GAGs at forming sites have been shown to be negative modulators of bone turnover (the most relevant tissue here would be TA1), negative modulators of mineral content, and also responsible for keeping the canalicular network free of mineral (most relevant tissue ages for the network statement would be TA3).

4. Pyridinoline enzymatic collagen cross-link content: F1-1 had very low values at all 3 tissue ages compared to healthy individuals. F2-1 had considerably less pronounced differences, although the values were also at the lower end or slightly below the healthy values.

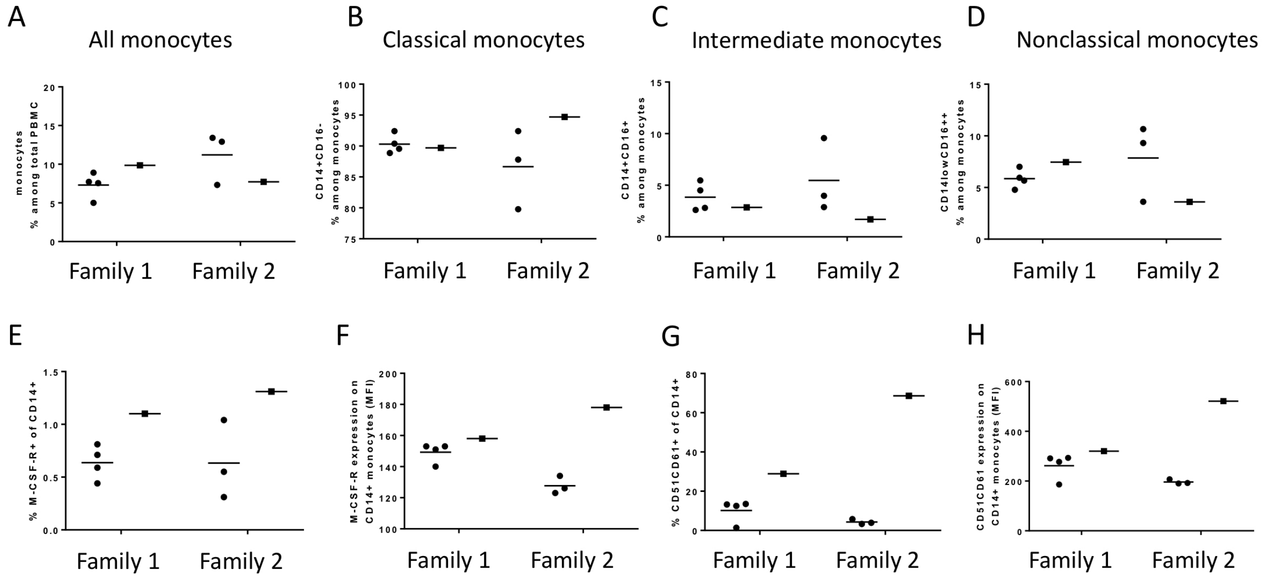

Figure S7. Analysis of osteoclast precursors in peripheral blood. Peripheral blood monocytes can be divided into subtypes based on their surface expression of CD14 and CD16: classical (CD14^{+}CD16^{-}), non-classical (CD14^{low}CD16^{+}) and intermediate (CD14^{+}CD16^{+}) monocytes. The classical CD14^{+}CD16^{-} monocytes are considered the major pool of osteoclast progenitors in peripheral blood. We analyzed different monocyte subpopulations by fluorescence-activated cell sorting (FACS) in Patient 1-1 (Family 1) and Patient 2-1 (Family 2) and compared to age and gender matched controls (C4-C7 and C1-3, respectively). Percentage of monocytes among total PBMC (A) and the proportion of classical (CD14^{+}CD16^{-}) (B), intermediate (CD14^{+}CD16^{+}) (C) and nonclassical (CD14^{low}CD16^{hi}) (D) monocytes was determined. The percentage of M-CSF-R+ (E) and CD51/CD61+ (F) among CD14+ monocytes was determined as well as the median fluorescent intensity (MFI) of the staining for M-CSF-R (F) and CD51/CD61 (H). Black squares, patients; black circles, controls.

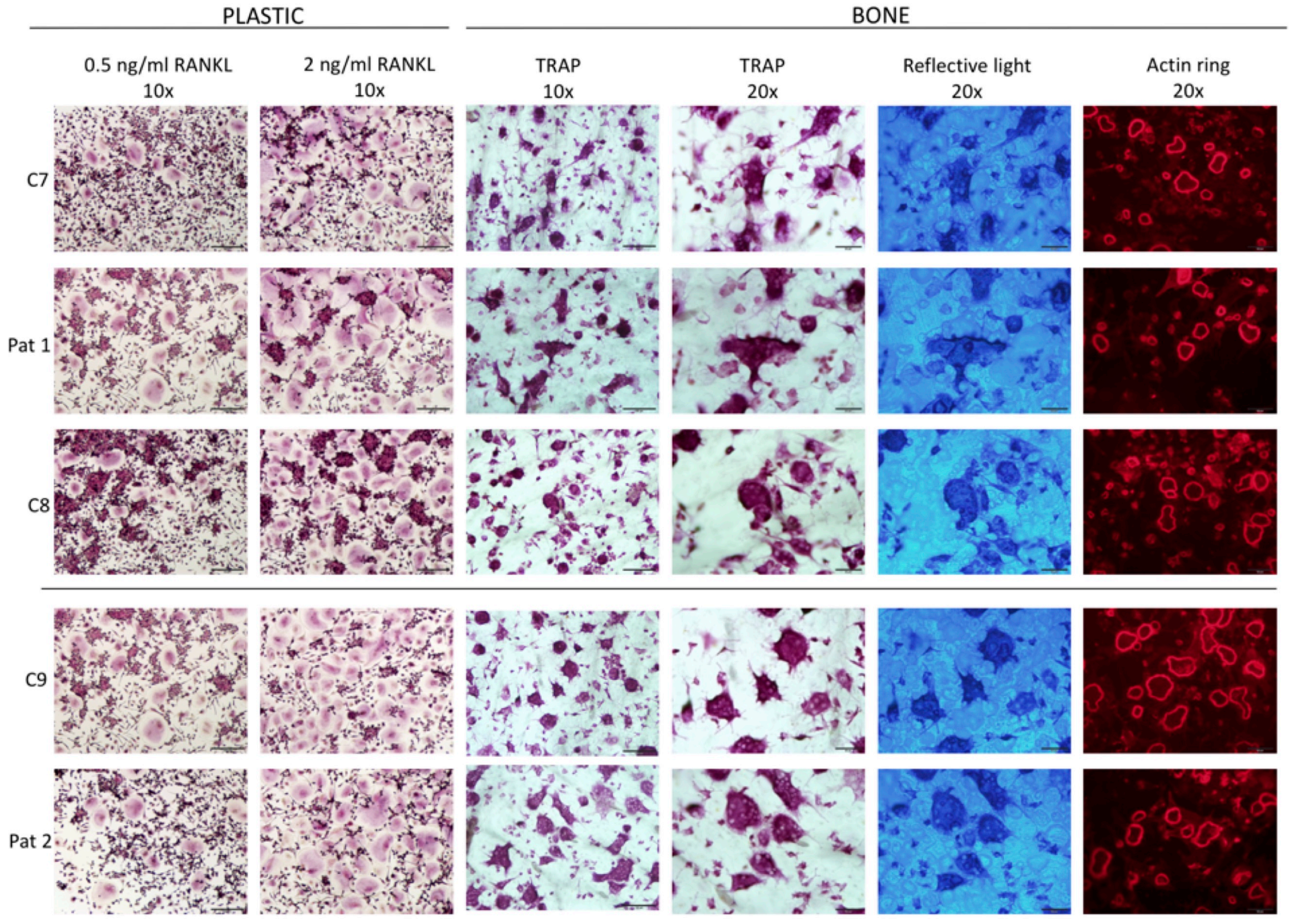

Figure S8. In vitro osteoclast differentiation on plastic and bone. CD14⁺ monocytes isolated from Patient F1-1 (Pat 1) and two age and gender matched controls (C7, C8) and Patient F2-1 (Pat 2) and one age and gender matched control (C9) were cultured in the presence of M-CSF and RANKL on plastic and on bone discs. Cultures on plastic were made in 0.5 (left) and 2 ng/ml RANKL (right) for 4 days before TRAP staining. Photos were taken with 10x objective and scale bar corresponds to 100 µm. Cultures on bone were made in 2 ng/ml RANKL for 8 days before TRAP staining. Photos were taken with 10x (scale bars 100 µm) and 20x objectives (scale bars 20 µm)**.** Photos were also taken using reflective light to visualize resorption pits (20x objective, scale bars 20 µm). At the same time point, parallel bone discs were stained with rhodamine conjugated phalloidin to visualize actin rings (20x objective, scale bars 20 µm). The *SGMS2* mutation did not affect osteoclast morphology on plastic or bone.

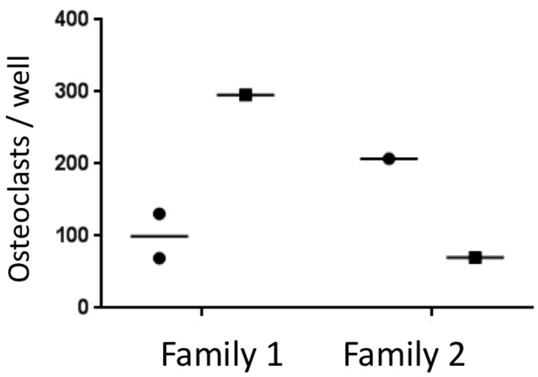

Figure S9. Number of osteoclasts formed on plastic. $CD14^+$ monocytes isolated from Patient F1-1 (Family 1) and two age and gender matched controls and Patient F2-1 (Family 2) and one age and gender matched control were cultured in the presence of M-CSF and RANKL (0.5 ng/ml) for 4 days before TRAP staining. For photos see Figure S8. Average number of TRAP positive multinucleated osteoclast per well was counted. Black squares, patients; black circles, controls. At sub-optimal concentration of RANKL (0.5 ng/ml), more osteoclasts were formed when the same number of monocytes were cultured from Patient F1-1 compared to two matched controls whereas monocytes from Patient F2-1 generated fewer osteoclasts on plastic compared to a matched control. At optimal concentration of RANKL (2 ng/ml) no obvious differences between osteoclasts from patients and healthy controls could be observed.

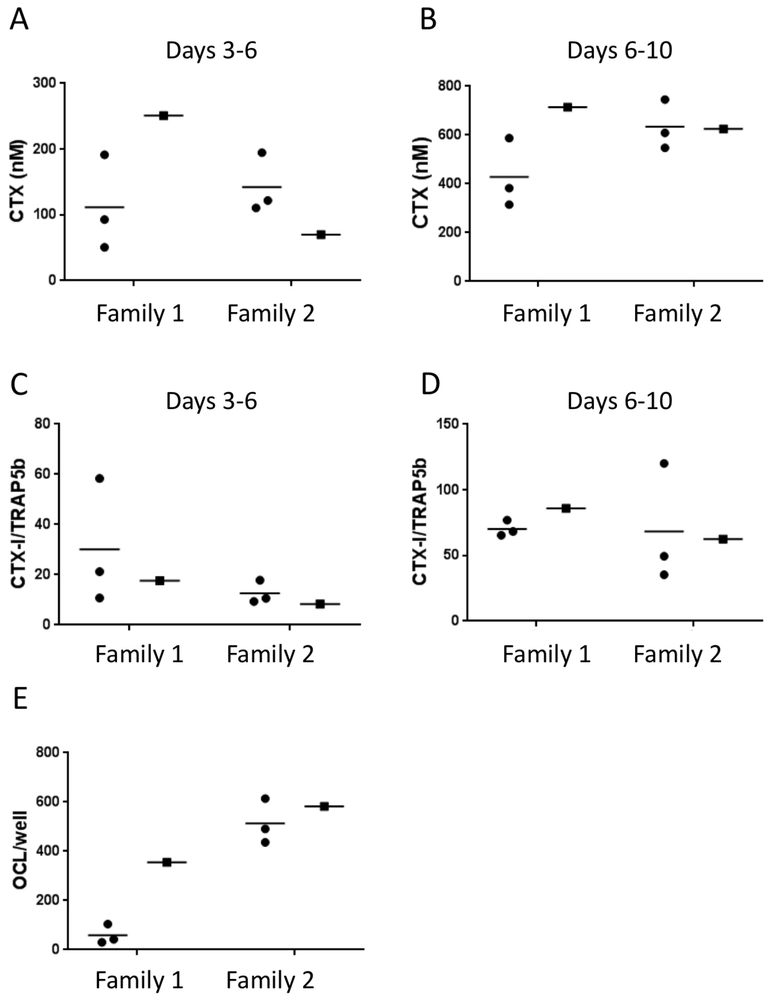

Figure S10. In vitro osteoclast bone resorption. CD14$^+$ monocytes isolated from Patient F1-1 (Family 1) and three age and gender matched controls (C5-C7) and Patient F2-1 (Family 2) and three age and gender matched controls (C1-3) were cultured in the presence of M-CSF and RANKL on bone discs. Resorptive capacity was measured as CTX-I released into the medium at days 3-6 (A) and days 6-10 (B) and as CTX-I/TRAP5b in culture medium at days 3-6 (C) and days 6-10 (D). Average number of TRAP positive multinucleated osteoclast per bone disc was counted (E). Black squares, patients; black circles, controls. In Patient F1-1, the resorptive capacity, measured as CTX-I, was higher at days 3-6 and 6-10 compared to the controls (A, B) while Patient F2-1 had slightly lower (days 3-6) or similar (days 6-10) resorptive capacity compared to controls (A, B). Both patients had similar CTX-I/TRAP5b ratio as their controls (C, D), indicating similar resorptive capacity per osteoclast. Increased resorption by Patient F1-1 osteoclasts was due to a higher number of osteoclasts on bone discs (E).

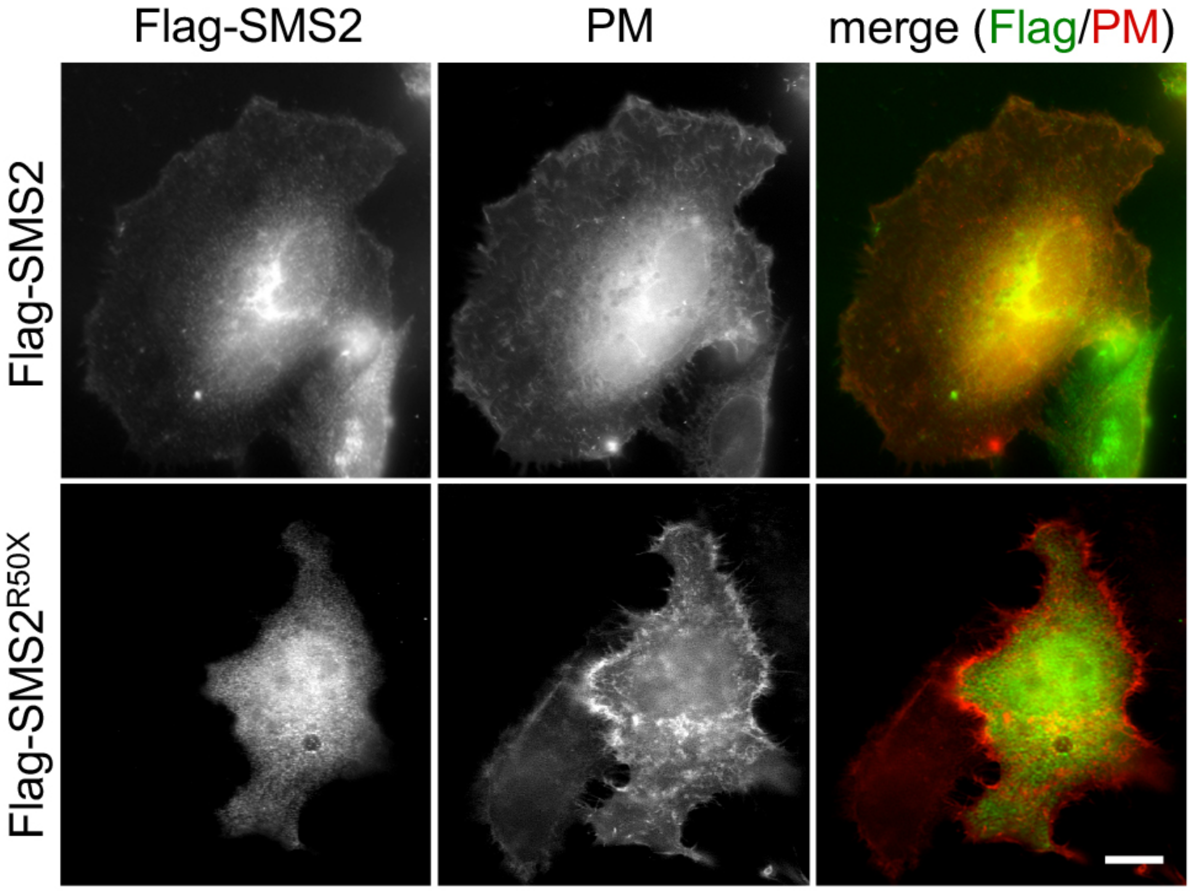

Figure S11. Subcellular distribution of FLAG-tagged SMS2 and SMS2^{R50X}. HeLa cells co-transfected with Flag-tagged SMS2 or SMS2^{R50X} and plasma membrane (PM) marker Lyn-FRB-mCherry (red) were stained with anti-Flag antibody (green) and then visualized by fluorescence microscopy. Bar, 10 µm.

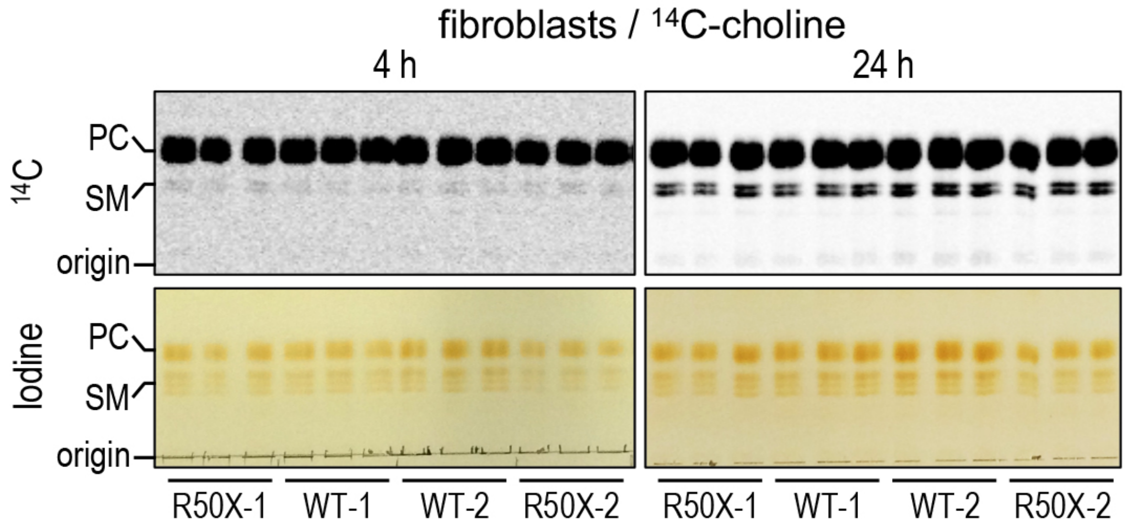

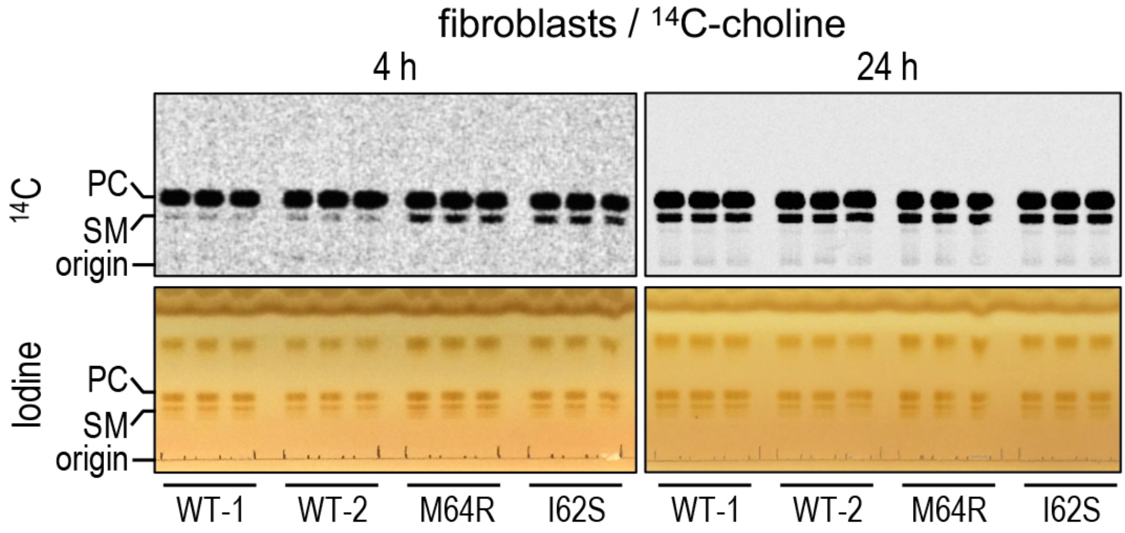

Figure S12. Metabolic labeling of patient-derived fibroblasts with C14-choline. TLC analysis of lipid extracts from control (WT) or patient-derived fibroblasts carrying heterozygous p.R50X (two patients), p.I62S or p.M64R mutations grown for 4 h or 24 h in medium supplemented with ^{14}C-choline. Incorporation of ^{14}C-choline into phosphocholine (PC) and sphingomyelin (SM) was analyzed by autoradiography (top). Lipids were stained with iodine vapor to verify that total lipid content between extracts was comparable (bottom). Note that part of the TLC analysis (bottom-row, left) is also shown in Figure 7C.

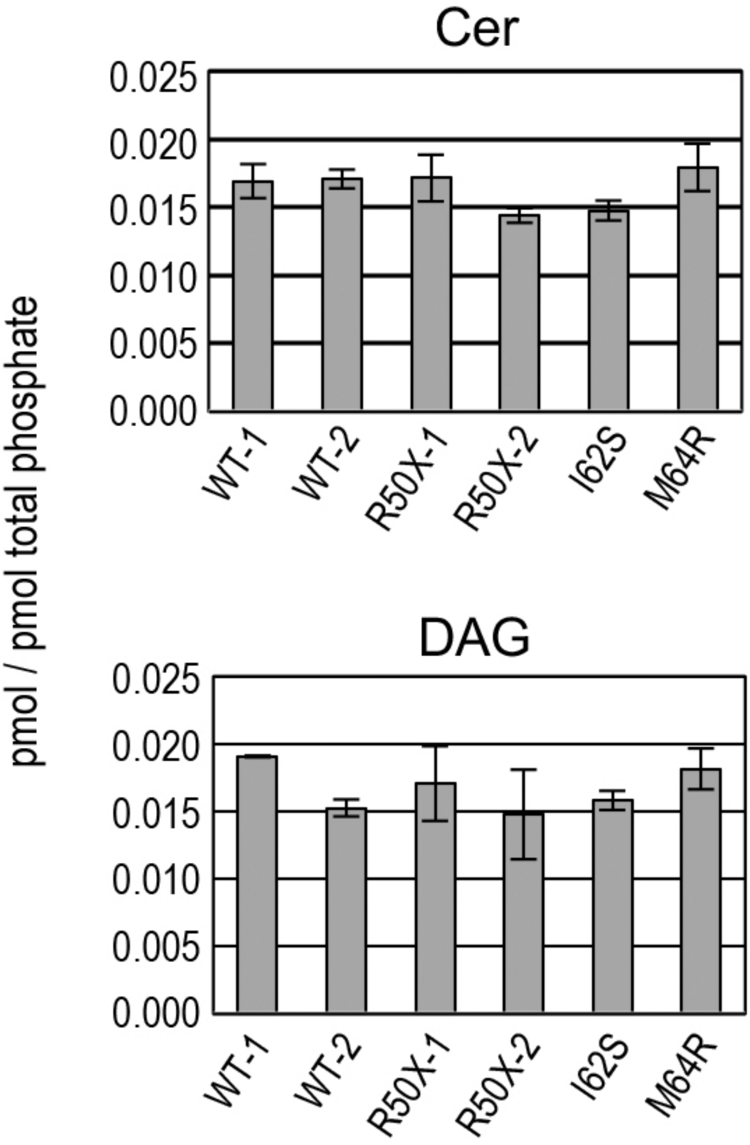

Figure S13. Liquid chromatography-mass spectrometry analysis of ceramide and diacylglycerol levels in patient-derived fibroblasts. Ceramide (Cer) and diacylglycerol (DAG) levels in total lipid extracts of control (WT) and patient-derived fibroblasts were determined by liquid chromatography-mass spectrometry and expressed as mole percent of total phospholipid analyzed. Data are means ± S.D.; n=3. R50X, I62S and M64R refer to the *SGMS2* mutation in the cell line.

3. SUPPLEMENTAL TABLES.

TABLE S1. Biochemical values in the affected individuals. P-Ca, plasma calcium; Pi, phosphate; 25-OHD, 25-hydroxy-vitamin D, U-Ca/U-Crea, urine calcium to creatinine ratio; PEA, phosphoetanolamine; B6-vit, vitamin B6. Supranormal values are marked in bold, subnormal values are marked with an asterix.

Subjects	Age (yrs)	P-Ca (mmol/l)	P-Pi (mmol/l)	P-PTH (ng/l)	S-25-OHD (nmol/l)	U-Ca/U-Crea (mmol/mmol)	U-PEA/Crea (µmol/mmol)	P-B6-vit (nmol/l)
F1-1	23	2.25	1.04	30	119	**0.76**	5	111
F1-2	60	1.26 #	0.79	58	94	0.26	3 *	60
F2-1	28	2.44	0.92	65	85	0.06	3 *	65
F3-1	7	2.35	1.32		102			
F3-2	8-10	2.38	1.39		80			
F3-4	35	2.45	1.07		165			
F4-1	6-7	2.45	1.45	20	145			
F5-1	38	2.27	0.82	37	53	<0.16	normal	60
F5-2	3	2.32	1.48	33	118	<0.40	normal	NA
F6-1	1-10	2.35	1.81	20	110			

Reference ranges: P-Ca (mmol/L) 19 mos – 5 yrs 2.15 – 2.7; 6 – 10 yrs, 2.05 – 2.70; >18 yrs 2.15 – 2.51; P-Pi (mmol/L) 2-12 yrs 1.2 – 1.8; males 18-49 yrs 0.71 – 1.53; females ≥ 18 yrs 0.76 – 1.41; males ≥ 50 yrs 0.71 – 1.23; P-PTH (ng/L) 15 – 70; S-25-OHD (nmol/L) >50; U-Ca/U-Crea (mmol/mmol) 3 – 5 yrs 0.05–1.1, 5 – 7 yrs 0.04–0.8, 7 – 18 yrs 0.04–0.7, >18 yrs < 0.7; U-PEA) (µmol/mmol Crea) 15–30 yrs 4.7–16.5, >45 yrs 5.4–10.5; P-B6-vitamin (nmol/L): all 20–120. #, Ionized calcium (mmol/L), ref. 1.1-1.3.

Table S2. Bone turnover markers in the affected individuals. P-ALP, plasma alkaline phosphatase; S-PINP, serum intact procollagen I N-terminal propeptide; ICTP, C-terminal type I collagen telopeptide; Ctx, C-terminal telopeptide of type I collagen; U-INTP, urine type I collagen cross-linked N-telopeptide. Reference ranges vary by age, sex and the diagnostic laboratory; appropriate reference ranges are given below each value. Supranormal values are marked in bold and subnormal values with an asterisk.

**Subject**	**Age (yrs)**	**P-ALP (U/L)**	**S-PINP**<br>(µg/L)	**S-Osteocalcin**<br>(µg/L)	**S-ICTP**<br>(µg/L)	**P-CTx**<br>(µg/L)	**U-Pyridinol**<br>(nmol/mmol Crea)	**U-Deoksipyridinol**<br>(nmol/mmol Crea)	**U-INTP**<br>(nmol/mmol Crea)
F1-1	23	70<br>(35-105)	51<br>(17-124)	**23**<br>(2-22)	**6.6**<br>(2.1-5.6)	0.33<br>(<0.57)			35<br>(<65)
F1-2	60	69<br>(35-105)	63<br>(17-124)	22<br>(2-22)	3.5<br>(2.1-5.0)	0.34<br>(<0.57)			31<br>(<65)
F2-1	28	**114**<br>(35-105)	73<br>(17-124)	**26**<br>(2-22)	5.0<br>(2.1-5.0)	**6.1**<br>(<0.57)			53<br>(<65)
F3-1	7	347<br>(150-440)					309<br>(117-325)	**99**<br>(20-75)	
F3-2	8-10	**448**<br>(150-440)					226<br>(81-267)	**71**<br>(13-61)	
F3-4	35	112<br>(40-120)					**91**<br>(20-40)	**30**<br>(5-11)	
F4-1	5-7	**412**<br>(125-320)			9.5<br>(7.0-16)		**453**<br>(117-325)	**197**<br>(20-75)	**>5650**<br>(201-626)
F5-1	38	98<br>(35-105)	25<br>(17-124)						
F5-2	3	230<br>(115-390)							
F6-1	10	**498**<br>(150-470)							

Table S3: Bone histomorphometry findings in transiliac bone biopsies.

Parameters	Control values *	Patient F1-1	Patient F2-1
Structural parameters			
**Bone volume/tissue volume BV/TV [%]**	24.4 ± 4.3	**25.13**	**19.11**
**Trabecular thickness Tb.Th [µm]**	148 ± 23	**124.05**	**111.80**
**Trabecular number Tb.N. [/mm]**	1.66 ± 0.22	**2.03**	**1.71**
**Cortical width Ct. Wi [mm]**	0.90 ± 0.33 #	**0.80**	**0.96**
Static parameters of bone formation			
**Osteoid volume/bone volume OV/BV [%]**	2.12 ± 1.0	**2.08**	**7.42**
**Osteoid thickness O.Th [µm]**	6.7 ± 1.7	**4.57**	**10.54**
**Osteoid surface/bone surface OS/BS [%]**	22.1 ± 7.8	**28.48**	**37.72**
**Osteoblast surface/bone surface Ob.S/BS [%]**	6.7 ± 4.5	**2.12**	**10.11**
Dynamic parameters of bone formation			
**Mineralizing surface MS/BS [%]**	11.07 ± 5.0	**10.12**	---
**Mineral apposition rate MAR [µm/d]**	0.87 ±0.09	**0.84**	---
**Adjusted apposition rate Aj. AR [µm/d]**	0.46 ± 0.10	**0.30**	---
**Bone formation rate/bone surface BFR/BS [µm/y]**	37.3 ± 16.7	**31.00**	---
**Bone formation rate/bone volume BFR/BV [%/y]**	49.9 ± 21.4	**52.84**	---
**Mineralization lag time Mlt [d]**	14.5 ± 3.00	**18.68**	---
Static parameters of bone resorption			
**Eroded surface/bone surface ES/BS [%]**	14.9 ± 5.6	**15.29**	**9.82**
**Osteoclast surface/bone surface Oc.S/BS [%]**	1.14 ± 0.74	**1.60**	**2.31**
**Osteoclast number N. Oc/ BS [/mm]**	0.29 ± 0.14	**0.55**	**0.56**

***** Published values from Glorieux et al. (17); # Mean value from two cortical plates;

--- tetracycline fluorescence intensities were too weak to be evaluated.

Table S4: BMDD findings in the transiliac bone biopsies of Patients F1-1 and F2-1.

	**Cancellous bone**					**Cortical bone**				
**BMDD variables**	**Patient F1-1**	**%Difference from Ref**	**Patient F2-1**	**%Difference vs Ref**	**Reference***	**Patient F1-1**	**%Difference from Ref**	**Patient F2-1**	**%Difference from Ref**	**Reference***
**CaMean [wt% Ca]**	**19.80**	-5.5	**19.18**	-8.4	20.95 (0.57)	**19.99**	-2.2	**18.70**	-8.6	20.45 [19.68; 21.04]
**CaPeak [wt% Ca]**	**20.97**	-3.2	**20.28**	-6.4	21.66 (0.52)	**21.84**	3.3	**19.76**	-6.5	21.14 [20.62; 21.75]
**CaWidth [Δwt% Ca]**	**5.37**	54.8	**5.03**	45.0	3.47 [3.12; 3.64]	**6.07**	59.3	**5.20**	36.5	3.81 [3.38; 4.38]
**CaLow [%]**	**16.57**	169.9	**19.85**	223.3	6.14 [4.90; 7.99]	**18.84**	107.9	**26.87**	196.6	9.06 [6.22; 15.00]
**CaHigh [%]**	**1.47**	65.2	**0.49**	-44.9	0.89 [0.43; 1.47]	**5.06**	1000.0	**1.09**	137.0	0.46 [0.28; 1.22]

*Reference: previously published values for cancellous and cortical bone in a young healthy reference cohort (18). Parameters and abbreviations are explained in Figure S5.

4. SUPPLEMENTAL REFERENCES

1. Gamsjaeger S, et al. Bone material properties in actively bone-forming trabeculae in postmenopausal women with osteoporosis after three years of treatment with once-yearly zoledronic acid. *J Bone Miner Res*. 2010;26(1):12-18.

2. Morris MD, Mandair GS. Raman Assessment of Bone Quality. *Clin Orthop Relat Res.* 2011;469(8):2160-2169.

3. Donnelly E, Chen DX, Boskey AL, Baker SP, van der Meulen MC. Contribution of Mineral to Bone Structural Behavior and Tissue Mechanical Properties. *Calcif Tissue Int.* 2010;87(5):450-460.

4. Paschalis EP, et al. Aging vs Postmenopausal Osteoporosis: Bone Composition and Maturation Kinetics at Actively Forming Trabecular Surfaces of Female Subjects Aged 1 to 84 Years Old, Journal of bone and mineral research. *J Bone Miner Res.* 2016; 31(2):347-357.

5. Gamsjaeger S, et al. Pediatric reference Raman data for material characteristics of iliac trabecular bone. *Bone.* 2014;69:89-97.

6. Granke M, Does MD, Nyman JS. The Role of Water Compartments in the Material Properties of Cortical Bone. *Calcif Tissue Int*. 2015;97(3):292-307.

7. Rodriguez-Florez N, et al. An investigation of the mineral in ductile and brittle cortical mouse bone. *J Bone Miner Res.* 2015;30(5):786-795.

8. Gamsjaeger S, Klaushofer K, Paschalis EP. Raman analysis of proteoglycans simultaneously in bone and cartilage. *J Raman Spectrosc*. 2014;45:794-800.

9. Paschalis EP, Gamsjaeger S, Tatakis DN, Hassler N, Robins SP, Klaushofer K. Fourier transform Infrared spectroscopic characterization of mineralizing type I collagen enzymatic trivalent cross-links. *Calcif Tissue Int.* 2015;96(1):18-29.

10. Garnero P. The contribution of collagen crosslinks to bone strength. *Bonekey Rep.* 2012;1:182.

11. Takeshita S, Kaji K, Kudo A. Identification and characterization of the new osteoclast progenitor with macrophage phenotypes being able to differentiate into mature osteoclasts. *J Bone Miner Res*. 2000;15(8):1477-88.

12. Granholm S, Lundberg P, Lerner UH. Calcitonin inhibits osteoclast formation in mouse haematopoetic cells independently of transcriptional regulation by receptor activator of NF-{kappa}B and c-Fms. *J Endocrinol*. 2007;195(3):415-27.

13. Bakker AD, Klein-Nulend J. Osteoblast isolation from murine calvaria and long bones. *Methods Mol Biol*. 2012;816:19-29.

14. Granholm S, Henning P, Lindholm C, Lerner UH. Osteoclast progenitor cells present in significant amounts in mouse calvarial osteoblast isolations and osteoclastogenesis increased by BMP-2. *Bone.* 2013;52(1):83-92.

15. Wishart DS, et al. HMDB 3.0—The Human Metabolome Database in 2013. *Nucleic Acids Res*. 2013;41(Database issue):D801–807.

16. Paschalis EP, et al. Aging Versus Postmenopausal Osteoporosis: Bone Composition and Maturation Kinetics at Actively-Forming Trabecular Surfaces of Female Subjects Aged 1 to 84 Years. *J Bone Miner Res.* 2016;31(2):347–57.

17. Glorieux FH, et al. Normative data for iliac bone histomorphometry in growing children. *Bone.* 2000;26(2):103-9.

18. Fratzl-Zelman N, et al. Normative data on mineralization density distribution in iliac bone biopsies of children, adolescents and young adults. *Bone.* 2009;44(6):1043-8.
